# Design and fabrication of a low-cost wireless camera imaging system for centrifugal microfluidics

**DOI:** 10.1016/j.ohx.2022.e00259

**Published:** 2022-01-08

**Authors:** Brian Regan, David Kinahan, Philip Daly, Richard O'Kennedy, David Collins

**Affiliations:** a School of Biotechnology, Dublin City University, Dublin 9, Ireland; b School of Mechanical Engineering, Dublin City University, Dublin 9, Ireland; c Hamad Bin Khalifa University, Qatar Foundation, Doha, Qatar

**Keywords:** Microfluidics, Imaging, Flow visualisation, Lab-on-a-Disc, Real-time measurements

## Abstract

Centrifugal microfluidic devices offer a robust method for low-volume fluid handling by combining low-cost instrumentation with highly integrated automation. Crucial to the efficacy of Lab-on-a-Disc (LoaD) device operation is the selection of robust valving technology, the design of on-disc fluidic structures, and accurate control of disc spin-speeds (centrifugal force) during operation. The design and refinement of fluidic and valving structures is often guided by inspecting disc operation using high-speed camera systems. This approach involves synchronising image acquisition with disc rotation to visualise liquid flow through a series of images often presented in a video format. Depending on the decisions taken, such systems can cost from €4,000 upwards. This paper outlines the development of a low-cost centrifugal test-stand with an integrated imaging system using a generic wireless camera to record videos directly to a smartphone device. This imaging system can be fabricated using only 3D printers and a low-cost CNC milling machine from widely available materials for approximately €350. High-fidelity imaging of the entire disc for flow visualisation and the recording of real-time colour intensity measurements are facilitated by this standalone device. A vibration analysis study has been performed to determine the rotational velocity range at which the system can be safely operated. Furthermore, the efficacy of the imaging system has been demonstrated by performing real-time colour intensity measurements of dyed water dilutions.

## Graphical abstract

**Figure:**
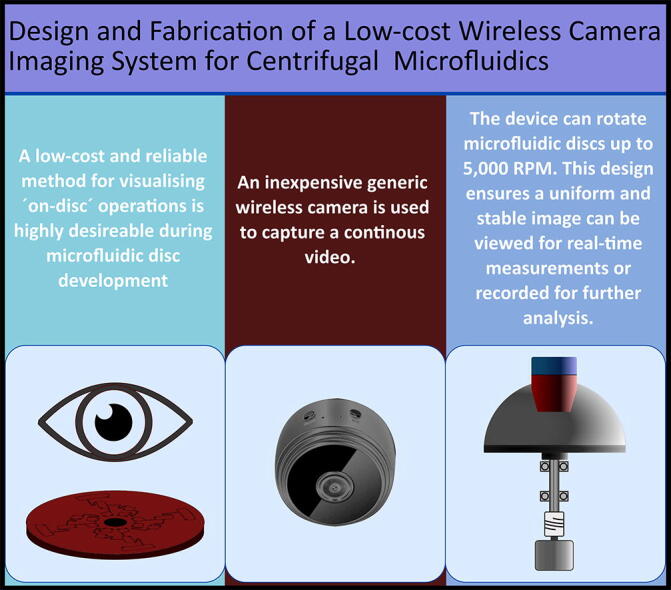

## Hardware in context

Microfluidic devices combine classical fluid dynamics at the macro-scale and dominant surface tension forces at the micro-scale to precisely manipulate small fluid volumes. This is highly beneficial when implementing sensing strategies as reagent consumption can be reduced and unit operations can be performed in parallel which in turn, decreases both analysis time and the cost of analyte detection. Hence, these devices find utility in a range of research and industrial areas such as biomedical applications [1], environmental monitoring [2] and food processing [3]. Microfluidic devices can contain various active and passive fluidic components such as micropumps, valves and micromixers to support enhanced control of on-chip fluids.

Centrifugal microfluidics, or Lab-on-a-Disc (LoaD), involves rotating a microfluidic chip about an axis to use the centrifugal force to manipulate fluids—or particles suspended in a fluid—which are present on the disc [1], [4], [5], [6], [7], [8], [9], [10]. These discs are typically, but not exclusively, designed in the same size as optical storage media (CD^TM^ or DVD^TM^) to potentially leverage the mass-manufacturing infrastructure associated with these products, and can also be operated on miniaturised instruments similar to a commercial Discman™ [11]. Applications can range from environmental and bioprocess monitoring [11], [12], [13], [14] to biomedical diagnostics including HIV detection [15],determination of circulating tumour cells [16], exosome isolation [17], nucleic acid identification [18] and use in immunoassays for rapid and automated analysis [19].

The use of the centrifugal force on LoaD devices has several advantages. The primary-one obviates the reliance on specialised microfluidic pumps as the rotational force, and so the pumping force, is controlled by the magnitude of the rotational velocity. Thus, the primary instrumentation is a simple and low-cost spindle motor. Another notable advantage is facile ‘world-to-chip’ interactions where the disc can be loaded with a pipette or syrette rather than needing specialised priming or bleeding of air [20]. The inherent centrifugal force is also a significant advantage for blood processing [21] and isolation of specific cohorts of white blood cells (WBCs) [22]. However, as all liquid on the disc is subjected to the centrifugal force during rotation, valving selection is critical; amongst valving technologies used are capillary burst valves [23], [24], siphon valves [25], dissolvable films [20], [26], membranes [27], [28], and laser-actuated valves [29], [30].

Imaging on a LoaD platform is typically achieved with a custom laboratory instrument generally referred to as a centrifugal test-stand [31]. In its typical form, this involves a controllable spindle motor which outputs a digital signal (from an encoder) as it passes a particular rotational position. This pulse is used to trigger an image from either a high-speed camera or to synchronize acquisition from a scientific camera with a strobe system [20]. This typically permits the acquisition of a series of images, or live video, where fluid movement can be clearly visualised, but the disc appears stationary. Hence, well-defined visualisation of ‘on-disc’ operations is highly beneficial when investigating dysfunctional fluidic structures or valves during disc development. Alongside this basic system, additional hardware can be integrated to enable actuation of specific valves based on laser ablation or IR heating [29], [30], a spring-balance for membrane valves [32], and a mechanical arm to vent specific chambers [33] or to activate membranes valves [34].

Many sensing techniques conducted on LoaD devices often employ transducers that produce colour changes [11] or the emittance of light upon photoexcitation [35] or following the generation of reaction products [30]. Measuring the intensity of these colourimetric or luminescent events often requires on-board reagents to be transferred to bench reader instruments. However, in many cases, test-stands can have additional components or modifications to avoid the need to move reagents off-disc. Applications can include high resolution imaging of cells [36] and facilitating sensing and automated / on-line measurement and biosensing [9]. Biosensing technologies which have been used, include fluorescence-based detection [37], surface plasmon resonance [38], absorbance [11], [39], and electrochemistry [40]. Recently, with the reduction in cost of electronic microprocessors, there is a growing trend towards developing customised systems which can rotate with the disc during sensing. Arduino^TM^ based systems [41] have been used for applications such as colour-sensing [42] and chemiluminescence [43]. Similarly, portable instruments have been modified and linked to the Lab-on-a-Disc for electrochemical-based sensing strategies [44], with one in particular employing a spy video camera for real-time viewing of on-disc operations [45].

Towards this trend of mating LoaD with co-rotating instrumentation, this paper describes the development of a low-cost wireless camera flow-visualisation system for ‘real-time’ ‘on-disc’ colour intensity measurements. This device incorporates a dome housing structure which is fastened to the spindle plate and contains a seated generic wireless camera to view and capture video footage directly to a smartphone device. As the entire camera assembly (dome housing, camera and circuit board housing containing the battery circuit board) rotates with the spindle plate, a highly stable image can be acquired and analysed for the measurement of any analyte. Moreover, this device is a cost-effective alternative to stationary high-speed camera systems for the development of centrifugal discs as it can easily allow the capture of video footage to assist in inspecting the efficacy of fluidic structures. The simplistic design and reliance on widely available fabrication techniques ensures this system can be easily reproduced at a low cost (≈ €350) and offers an alternative to traditional stationary high-speed camera systems for disc development and bespoke reader systems for ‘on-disc’ analyte detection.

## Hardware in description

**Fig. 1:**
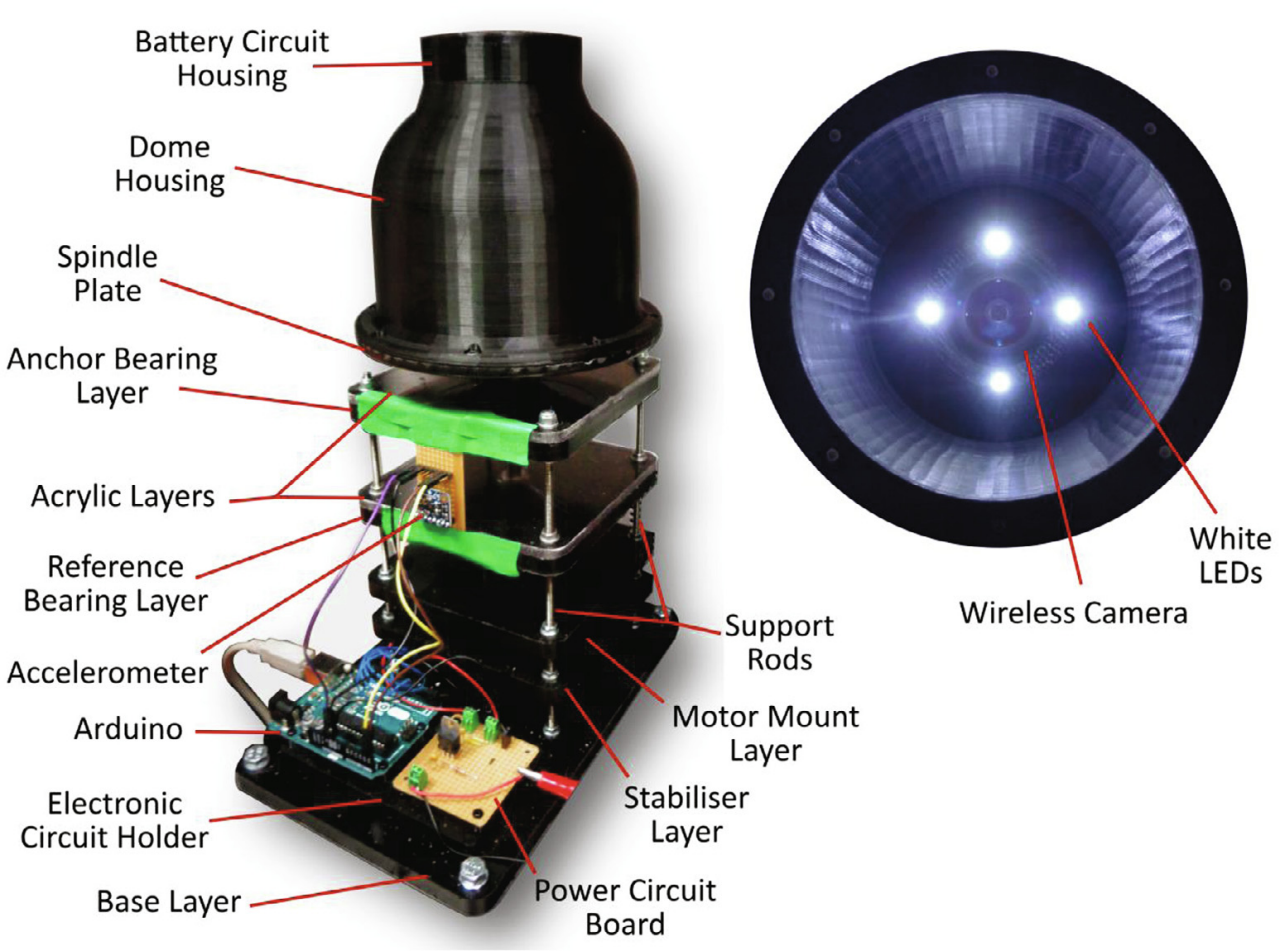
[Left] Wireless camera imaging device with dome housing structure mounted to the spindle stage and the battery circuit board housing is secured to the housing structure. An Arduino^TM^ Uno microcontroller is shown beside the power circuit board. An accelerometer (MMA8451 Adafruit breakout) is taped to the bearing layers which is subsequently used for the vibration analysis. [Right] The inside of the dome housing structure shown with illuminated white LEDs and the wireless camera installed.

In contrast to existing traditional imaging systems used for the visualisation of centrifugal discs, this device has been designed as a standalone unit as depicted in Fig. 1. It is important to note, that although this device is a standalone unit; it should not be operated without a protective shield or enclosure for the safety of the operator. A protective shield has intentionally been omitted from Fig. 1. The wireless camera indicated below is seated directly in the dome housing structure and is bound within the seat by the battery circuit housing which is mounted directly above it. The dome housing is secured to the spindle plate and thus, the entire camera assembly rotates in unity with the spindle plate and the disc that it views, which in turn, ensures the video footage acquired is highly stable. As the dome housing effectively prevents ambient light from entering the unit, 4 white LEDs are used to illuminate the surface of the disc. These LEDs are powered by coin cell batteries in the battery circuit contained in the circuit housing above.

The wireless camera imaging system is constructed as a multi-layered platform with each acetal and acrylic layer supporting a function. These layers were fabricated using an EVO II CNC milling machine (MakerDreams) in which the features and contours of the layers were cut from segments of acetal and acrylic sheets. A description of each layer is outlined below:

• Base: Fabricated from machined 8 mm acetal, this piece is mounted to an optical table and secured using hex bolts. The support rods are inserted into threaded holes and fastened using lock nuts located in hole cavities concentric to the through holes. The electronic circuit holder, which the microcontroller and power circuit is mounted upon, is also secured to the base.
• Stabiliser: This is a layer of acetal in which the brushless DC (BLDC) motor is inserted. During periods of vibrations, this layer acts to minimise movement of the motor.
• Motor mount: The motor is securely attached to this layer using nuts and bolts.
• Reference Bearing: A radial bearing used to support the spindle shaft is pressed into this layer.
• Acrylic: Constructed from a 5 mm acrylic sheet segment, this layer is machined and clamped to the acetal piece to bound the bearing.
• Anchor Bearing: This is identical to the reference bearing layer. The support rods protrude through the top of this layer and are fastened using lock nuts.

### Camera specifications and positioning

**Fig. 2:**
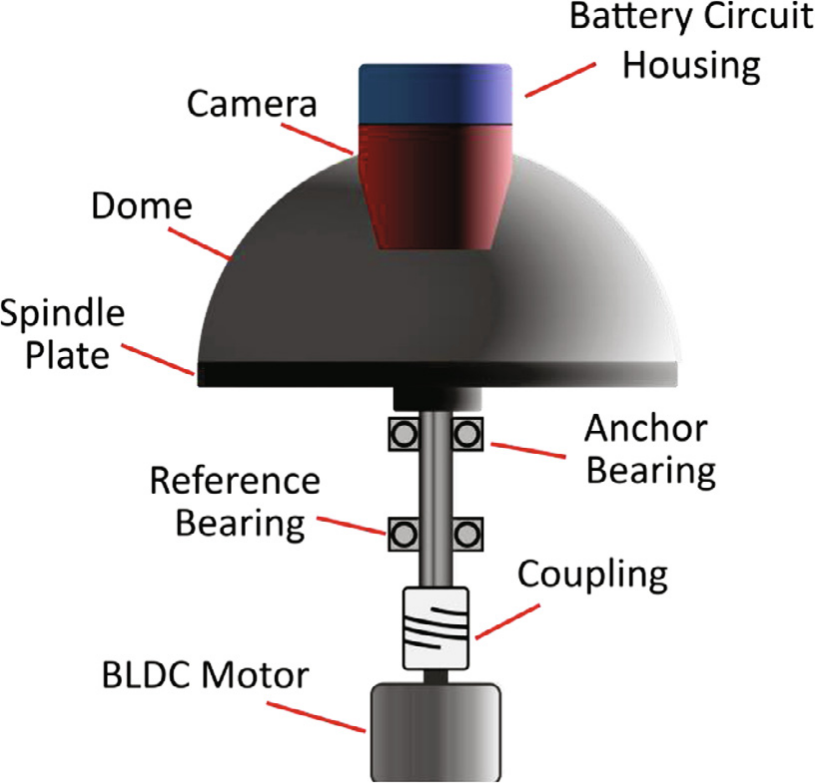
Simplified representation of the wireless camera imaging system.

The wireless camera that is used in the system is advertised as a generic spy camera (Aobo HC005) and is widely available from various e-commerce platforms. Despite many of these cameras appearing identical in design, they generally possess differing specifications. The advantage of using a generic camera is that for certain requirements, cameras with different specifications might be favoured and thus, cameras can easily be exchanged for inexpensive replacements. In the context of this system—which was designed to facilitate high-quality visualisation of liquid flow on LoaD devices during high velocity operation— considerable emphasis was placed upon the camera field-of-view (FOV) and the focal distance. To minimise the inertial forces generated during device operation, the camera should be positioned near the surface of the spindle plate, or more precisely, at a minimum distance from the anchor bearing, as shown in Fig. 2. Any imbalances and misalignments in the system will be amplified the further the camera is located from the anchor bearing. Hence, acquiring a camera with a short focal distance and a wide FOV is imperative for enhancing the performance of the system and minimising device vibrations.

The specifications of the camera acquired (Aoba HC005) permitted placement approximately 85 mm from the spindle plate surface to provide an in-focus image whilst viewing the majority of the 120 mm diameter disc surface. The FOV was calculated to be approximately 110°, however, the smartphone app used to view the received footage (P2PLiveCam) produces a rectangular display, hence, the actual FOV may be slightly wider. The smartphone app is free to download and stores the recorded videos in an MP4 file format directly to the smartphone memory storage. The wireless camera generates its own network and can transmit data directly to the smartphone device without access to a shared WIFI network.

### Dome housing and spindle plate

**Fig. 3:**
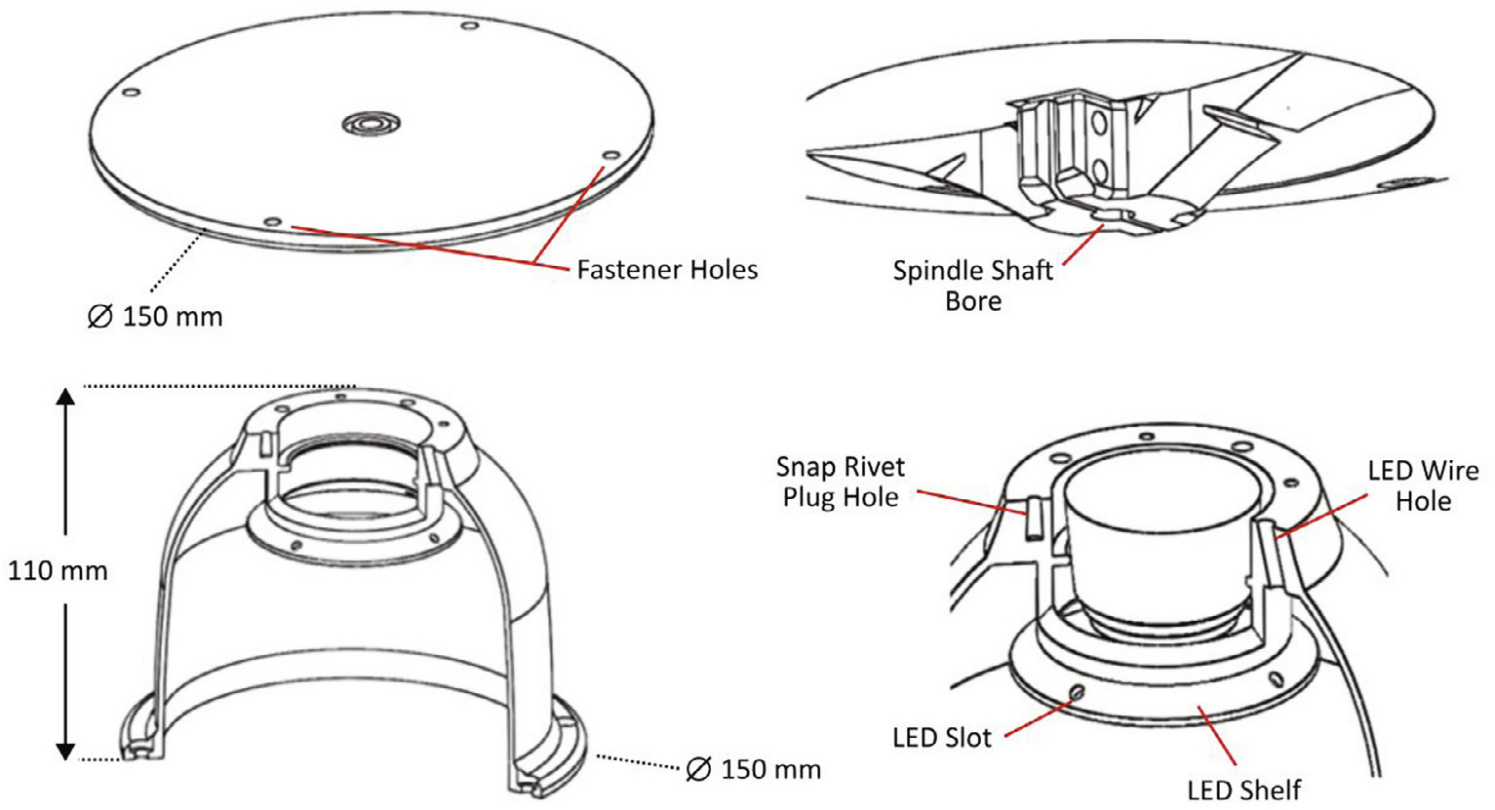
Spindle plate and dome housing structure with selected dimensions and relevant features illustrated. [Top left] The top surface of the spindle plate on which the LoaD is placed upon and the dome structure is secured. [Top right] The spindle shaft clamp on the underside of the spindle plate. (Bottom left] Section view of the dome housing structure with the LED shelf exposed. [Bottom right] Detailed section of the dome housing structure with the wireless camera installed. The sectioned view pierces a hole that the LED wires are fed and a snap rivet plug hole.

Fundamental to the operation of this device is the ability to securely position the wireless camera directly above the rotating disc. Moreover, preventing image disturbance due to ambient lighting exposure ensures a uniform video can be captured and real-time analyte measurement can be achieved. The bespoke design of the dome housing structure, which is shown in Fig. 3, fulfils both of these critical criteria. To accommodate the printing of the dome housing structure, the 3D printing build volume must be quite large (160 × 160 × 110 mm), hence a Qidi Tech X-MAX FDM 3D printer—which has a build volume of 300 × 250 × 300 mm—is used. The top section of the dome is specifically designed so that the camera is seated flush with the flat top surface and is retained in position by fastening the battery circuit board housing directly to the top surface using plastic snap rivets. The soft outer body of the camera acts somewhat as a spring by allowing the camera to be pressed below the plane of the top surface and then providing a reacting force towards the bottom surface of the circuit housing. A shelf is incorporated into the dome design to hold LEDs which are press fitted into holes. The power wires for the LEDs run through holes in the dome structure and into openings in the circuit housing.

During spinning, the dome housing is secured to the spindle plate using mechanical fasteners. The spindle plate, which is depicted in Fig. 3, was fabricated using a Stratasys Objet260 Connex 1 stereolithography (SLA) 3D printer. The spindle plate is a solid mass and weighs 182.5 g which is heavier than the dome (136.5 g), but lighter than the camera assembly (202.5 g). Nonetheless, the solid body of the spindle plate ensures a relatively large mass is positioned near the anchor bearing. The spindle plate was originally printed using the FDM printer (Qidi Tech X-Max), however, the dimensional accuracy of the bore was inadequate and resulted in a slight misalignment which generated excessive vibrations. Furthermore, the rigidity and strength of the FDM printed spindle plate, even after applying a 100 % infill, was inferior to that printed using the SLA 3D printer. A higher quality FDM 3D printer might be capable of producing a spindle plate of improved accuracy and strength.

### Spindle control

Many centrifugal test-stand platforms utilise servo or stepper motors to rotate the spindle plate. These systems can require expensive motor drivers and can be quite bulky in size, hence, to minimise the costs and simplify the overall system design, a BLDC motor (NMB Technologies Corp. DIA42B20W32A) is used to rotate the spindle in this system. The motor acquired has inbuilt encoders and a motor driver that is configured for PWM control which is generated by an Arduino^TM^ Uno microcontroller. An Arduino^TM^ program script (Wifi_Camera_System_Control) was written to control the spindle rotation which includes a PI controller for speed regulation. The PI controller calculates the duty cycle of the PWM signal by sampling the measured velocity and comparing it with the set velocity every 100 ms. The measured velocity is updated every 100 ms by determining the number of encoder pulses within this time period—considering 100 pulses are produced per revolution. This controller has been specifically tuned to minimise overshoots which is particularly pertinent for microfluidic disc designs that incorporate speed-regulated valving structures. The program script also includes unidirectional and bidirectional mixing functions, the latter of which requires the signal on the BLDC motor direction pin to be repeatedly inverted.

## Design files

**Table 1:** Design files containing models of system components for fabrication and assembly. Includes generic CNC files, program scripts and circuit schematics.

**Design File Name**	**File Type**	**Open Source License**	**File Location**
*Circuit Board Holder*	CAD and STL	*CC-BY 4.0*	https://doi.org/10.17632/5rrc89f2kj.1
*Battery Circuit Housing*	CAD and STL	*CC-BY 4.0*	https://doi.org/10.17632/5rrc89f2kj.1
*Battery Circuit Housing Lid*	CAD and STL	*CC-BY 4.0*	https://doi.org/10.17632/5rrc89f2kj.1
*Spindle Plate Ring*	CAD and STL	*CC-BY 4.0*	https://doi.org/10.17632/5rrc89f2kj.1
*Dome Housing Structure*	CAD and STL	*CC-BY 4.0*	https://doi.org/10.17632/5rrc89f2kj.1
*Spindle Plate*	CAD and STL	*CC-BY 4.0*	https://doi.org/10.17632/5rrc89f2kj.1
*Acrylic Layer*	CAD and NC	*CC-BY 4.0*	https://doi.org/10.17632/5rrc89f2kj.1
*Anchor Bearing Layer*	CAD and NC	*CC-BY 4.0*	https://doi.org/10.17632/5rrc89f2kj.1
*Motor Mount Layer*	CAD and NC	*CC-BY 4.0*	https://doi.org/10.17632/5rrc89f2kj.1
*Stabiliser Layer*	CAD and NC	*CC-BY 4.0*	https://doi.org/10.17632/5rrc89f2kj.1
*Acetal Base*	CAD and NC	*CC-BY 4.0*	https://doi.org/10.17632/5rrc89f2kj.1
*Circular Circuit Board*	CAD and NC	*CC-BY 4.0*	https://doi.org/10.17632/5rrc89f2kj.1
*Spindle Shaft*	CAD and PDF (Drawing)	*CC-BY 4.0*	https://doi.org/10.17632/5rrc89f2kj.1
*Generic Wireless Camera Casing*	CAD	*CC-BY 4.0*	https://doi.org/10.17632/5rrc89f2kj.1
*Brushless DC Motor*	CAD	*CC-BY 4.0*	https://doi.org/10.17632/5rrc89f2kj.1
*Radial Ball Bearing*	CAD	*CC-BY 4.0*	https://doi.org/10.17632/5rrc89f2kj.1
*Support Rod*	CAD	*CC-BY 4.0*	https://doi.org/10.17632/5rrc89f2kj.1
*Wireless Camera Imaging System*	CAD	*CC-BY 4.0*	https://doi.org/10.17632/5rrc89f2kj.1
*Wifi_Camera_System_Control*	INO	*CC-BY 4.0*	https://doi.org/10.17632/5rrc89f2kj.1
*Wifi_Camera_System_Control_ with_Vibration*	INO	*CC-BY 4.0*	https://doi.org/10.17632/5rrc89f2kj.1
*Power Circuit Schematic*	PNG	*CC-BY 4.0*	Supplementary Data 1
*Battery Circuit Schematic*	PNG	*CC-BY 4.0*	Supplementary Data 1

STL files only are supplied for 3D printed models.

Numerical code (NC) files contain CNC toolpath instructions for machining parts. These have been generated using a Grbl post processor in Autodesk’s Fusion 360. These NC files may not be compatible with certain CNC milling machines.

Only the anchor bearing layer model is provided as it is identical to the reference bearing layer.

The complete assembly has been supplied to help visualise the exact location of components for physical device assembly.

## Bill of materials

**Table 2:** Complete bill of materials (BOM) for construction of the wireless camera imaging system.

***Materials and Mechanical Components***						
**Vendor Part Number**	**Component**	**Number**	**Cost per Unit (€)**	**Cost (€)**	**Source of Materials**	**Material Type**
282–0171	*Stabiliser Layer*	1	6.48	6.48	Radionics Ireland	Acetal
282–0171	*Motor Mount Layer*	1	6.48	6.48	Radionics Ireland	Acetal
282–0171	*Anchor Bearing Layer*	1	6.48	6.48	Radionics Ireland	Acetal
282–0171	*Reference Bearing Layer*	1	6.48	6.48	Radionics Ireland	Acetal
282–0171	*Base Layer*	1	12.97	12.97	Radionics Ireland	Acetal
824–525	*Acrylic Layer*	2	3.87	7.73	Radionics Ireland	Acrylic
467–9818	*Socket Screw (M3)*	4	0.11	0.44	Radionics Ireland	Steel
524–281	*Lock Nut (M3)*	4	0.05	0.21	Radionics Ireland	Steel
560–338	*Plain Washer (M3)*	8	0.01	0.06	Radionics	Steel
530–309	*Support Rod (M4)*	4	0.64	2.55	Radionics Ireland	Steel
525–896	*Hex Nut (M4)*	36	0.02	0.72	Radionics Ireland	Steel
524–304	*Lock Nut (M4)*	8	0.06	0.44	Radionics Ireland	Steel
525–925	*Plain Washer (M4)*	44	0.01	0.52	Radionics Ireland	Steel
2,506,053	*Spring Washer (M4)*	2	0.02	0.04	Farnell Components (Ire)	Stainless Steel
281–237	*12 mm Socket Screw (M4)*	4	0.15	0.58	Radionics Ireland	Steel
281–243	*16 mm Socket Screw (M4)*	2	0.17	0.34	Radionics Ireland	Steel
917–3107	*Hex Bolt (M6)*	6	0.20	1.19	Radionics Ireland	Steel
525–947	*Plain Washer (M6)*	6	0.02	0.11	Radionics Ireland	Steel
527–612	*Hex Nut (M8)*	1	0.09	0.09	Radionics Ireland	Steel
530–797	*Plain Washer (M8)*	1	0.06	0.06	Radionics Ireland	Steel
122–4389	*Plain Washer (M10)*	1	0.04	0.04	Radionics Ireland	Steel
477–9895	*Shaft Coupling (8 mm − 6 mm)*	1	44.12	44.12	Radionics Ireland	Aluminium
612–5903	*Radial Ball Bearing*	2	5.77	11.54	Radionics Ireland	Steel
NA	*Spindle Shaft*	1	34.44	34.44	Parsec Precision Tooling	Steel
2,914,448	*Snap Rivet*	16	0.09	1.39	Farnell Components (Ire)	Nylon
P121378-ND	*Brushless DC Motor*	1	37.92	37.92	Digikey Electronics	Electro-mechanical
***Electronics***						
**Vendor Part Number**	**Component**	**Number**	**Cost per Unit (€)**	**Cost (€)**	**Source of Materials**	**Material Type**
715–4081	Arduino^TM^*Uno Microcontroller*	1	24.17	24.17	Radionics Ireland	Electronic
NA	*Wireless Camera*	1	18.07	18.07	Ebay	Electronic
713–3949	*White LED*	4	1.00	4.00	Radionics Ireland	Electronic
1,668,331	*PCB Receptacle*	1	0.40	0.40	Farnell Components (Ire)	BLCP
790–1098	*PCB Terminal*	5	0.69	3.46	Radionics Ireland	Polyamide
913–9065	*Slide Switch*	2	1.51	3.02	Radionics Ireland	Polyamide
785–6762	*Voltage Regulator (5 V)*	1	1.09	1.09	Radionics Ireland	Electronic
1,536,938	*Matrix Board*	1	4.38	4.38	Farnell Components (Ire)	Epoxy Paper
191–6784	*Coin Cell*	2	1.32	2.64	Radionics Ireland	Lithium
2,860,100	*Ceramic Capacitor (*0.1 µ*F)*	1	0.34	0.34	Farnell Components (Ire)	Ceramic
2,309,021	*Ceramic Capacitor (*0.33 µ*F)*	1	0.30	0.30	Farnell Components (Ire)	Ceramic
267–1777	1kΩ Resistor	1	0.13	0.13	Radionics	Ceramic
C161656	*BLDC Connector Housing*	1	0.07	0.07	LCSC Electronics	PBT
C595051	*BLDC Connector Crimp Terminal*	8	0.01	0.08	LCSC Electronics	Phosphor bronze, tin-plated
905–4665	Accelerometer*	1	8.48	8.48	Radionics Ireland	Electronic
718–9674	8-pin Header*	1	0.77	0.77	Radionics Ireland	Phosphor bronze
481–1775	Heat Shrink	8	0.05	0.40	Radionics Ireland	Polyolefin
2,290,848	*120 mm Red Wire (22 AWG)*	4	0.05	0.06	Farnell Components (Ire)	Tinned Copper
2,290,852	*120 mm Black Wire (22 AWG)*	4	0.06	0.14	Farnell Components (Ire)	Tinned Copper
872–4464	*170 mm Blue Wire (26 AWG)*	5	0.03	0.14	Radionics Ireland	Tinned Copper
1,764,904	*170 mm Black Wire (26 AWG)*	1	0.10	0.11	Farnell Components (Ire)	Tinned Copper
1,764,910	*170 mm Red Wire (26 AWG)*	2	0.10	0.22	Farnell Components (Ire)	Tinned Copper
1,764,904	100 mm Black Wire (26 AWG)	4	0.06	0.26	Farnell Components (Ire)	Tinned Copper
1,764,910	100 mm Red Wire (26 AWG)	4	0.06	0.26	Farnell Components (Ire)	Tinned Copper
***3D Printed Parts***						
**Vendor Part Number**	**Component**	**Number**	**Cost per Unit (€)**	**Cost (€)(0.04 €/g)**	**Source of Materials**	**Material Type**
183–0251	*Circuit Board Holder*	1	0.71	0.71	Radionics Ireland	PLA
183–0251	*Battery Circuit Housing*	1	0.62	0.62	Radionics Ireland	PLA
183–0251	*Battery Circuit Housing Lid*	1	0.40	0.40	Radionics Ireland	PLA
183–0251	*Spindle Plate Ring*	1	0.04	0.04	Radionics Ireland	PLA
183–0251	*Dome Housing Structure*	1	9.91	9.91	Radionics Ireland	PLA
NA	*Spindle Plate*	1	76.33	76.33	Stratasys	Vero Black™
***Total Cost: €344.40***						

* The accelerometer and associated components are not required for the operation of the device.

The FDM 3D printed parts were created from PLA acquired from Radionics Ireland. Costs of individual parts have been calculated from the price of the PLA filament reel.

BLCP - Black liquid crystal polymer, PBT - Polybutylene terephthalate, PLA - Polylactic Acid.

## Build instructions

As several parts of this device are created in-house, such as those produced using CNC machining and 3D printing, the construction of this device can be divided into two distinct stages: component fabrication and assembly (Table 2).

### Component fabrication

This section primarily details the materials processing methods implemented to fabricate components used in the construction of the wireless camera imaging system. The crimping technique used to create the BLDC cable connector assembly is also outlined.

#### Machining platform layers

1. Cut the acetal and acrylic sheets into segments using a power saw tool. An example of acetal segment allocation is shown in Fig. 4a. The acrylic layer segments can also be cut from the acrylic sheet using a power saw.

**Fig. 4:**
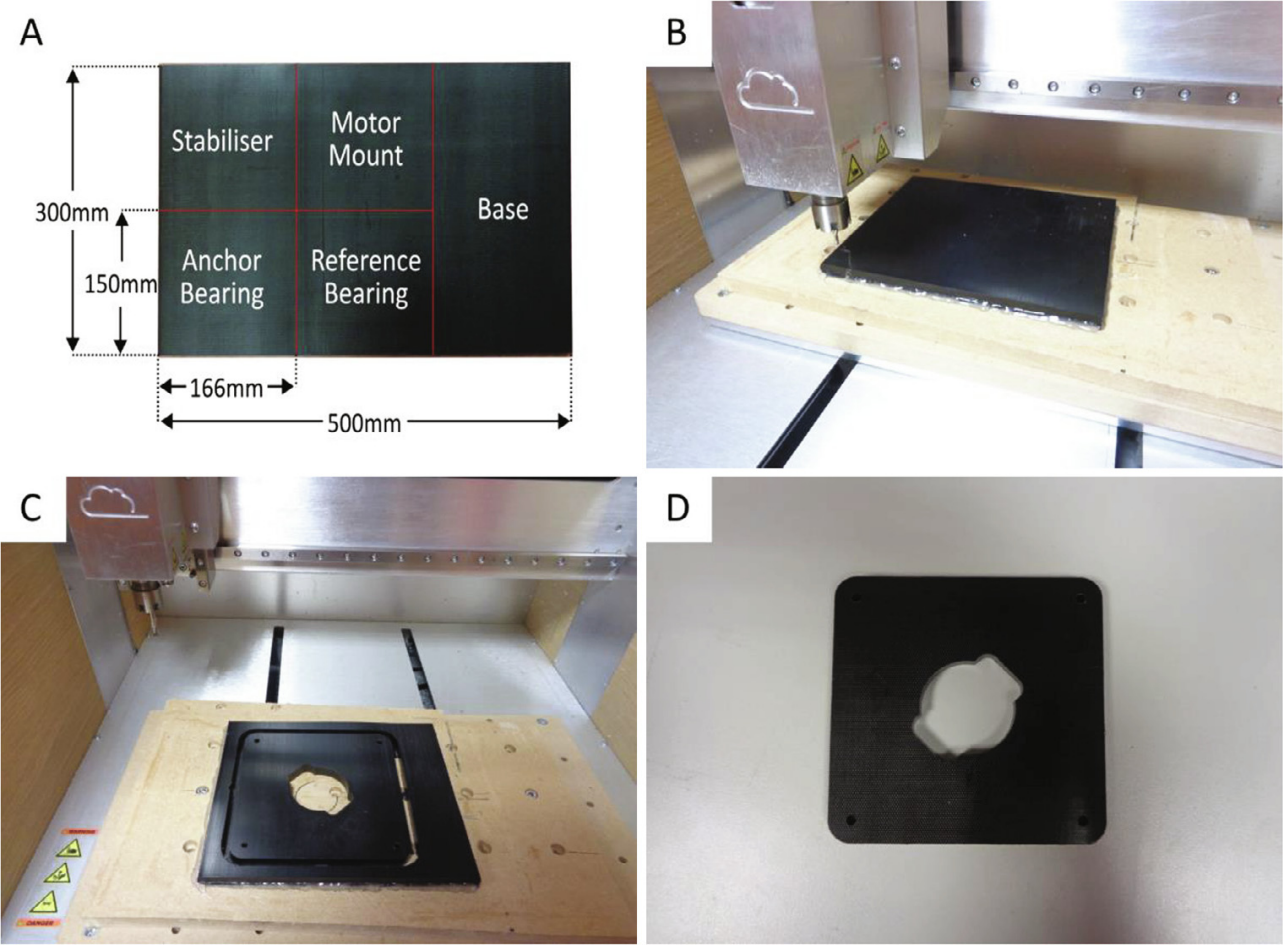
Machining the stabiliser layer from an acetal sheet segment. (A) Individual segments of acetal sheet are allocated to specific platform layers. The red markings indicate approximate cutting lines and the dimensions of the individual segments are also illustrated. (B) A segment of the acetal sheet secured on the CNC milling table by applying hot glue to the edges of the piece. The cutting tool is positioned at the origin of work coordinate system. C) The stabiliser layer secured to the CNC table immediately after features and the contour have been cut from the acetal segment. (D) The stabiliser layer after edges have been cleaned using a scalpel and a file. (For interpretation of the references to colour in this figure legend, the reader is referred to the web version of this article.)
2. Clamp a segment to the CNC machine table. This can be achieved using suitable clamping fixtures or by applying an adhesive such as hot glue.
3. Set up the CNC machine accordingly and upload the NC file. All the NC files have been generated with the working origin located at the bottom left of each toolpath. For the EVO II CNC machine, the working origin is initialised at the bottom left corner of the acetal segment as depicted in Fig. 4b.
4. Two separate NC files have been generated for each acetal and acrylic layer as a different (3 mm for boring & 5 mm for all operations) cutting tool (standard flat bottom milling cutter) is used for each operation. Importantly, the “All Operations” instruction should be performed last as this includes machining of the layer contour. Tabs have been included in the tool path, however, to ensure the piece remains secured to the table during cutting. Ensure the flute length of the tool used exceeds 9 mm for the acetal layers. These layers are approximately 8.8 mm in thickness which is slightly greater than that stated in the supplier’s product description (8 mm).
5. After cutting has ceased, carefully remove the piece from the machine. Use snips to remove the layer from the acetal segment and clean up the edges using a file or/and a scalpel. Ensure all material has been cleared from the through holes. The completed stabiliser layer is depicted in Fig. 4d.

The above process is applied to all of the platform layers. After machining the acetal base layer, the four through holes which the support rods are inserted are threaded using an M4 tapping tool. Ensure loose material is removed from these holes after threading. Additionally, the radial ball bearings should be pressed into the anchor and reference bearing layers prior to assembly.

#### Circuit boards and connectors

**Fig. 5:**
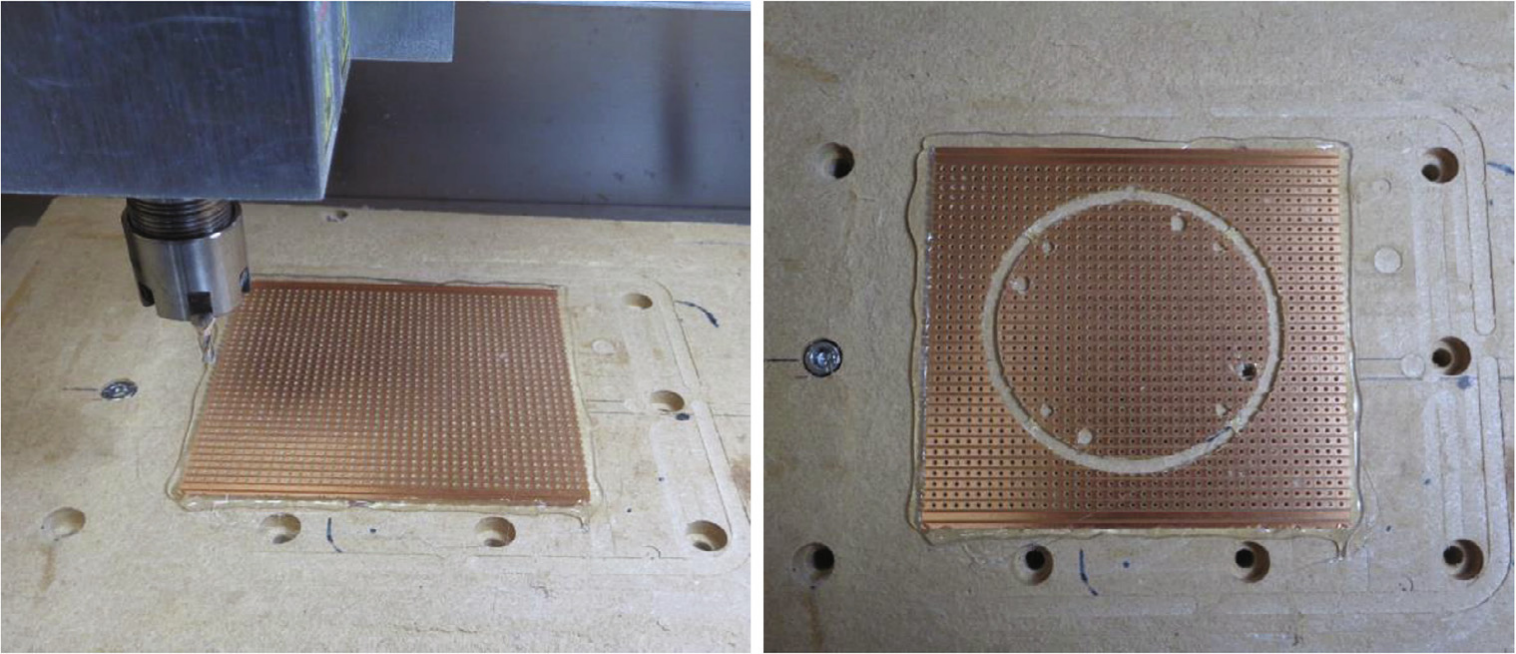
Machining the circular circuit board. The matrix board is secured to the milling table using hot glue and the features cut into this are visible on the right after machining.

The procedure described above outlining the machining of the acetal layers can be followed when cutting the circular circuit board from the matrix board. Similar to the acetal segment used to fabricate the stabiliser layer, the matrix board can also be secured to the milling table using hot glue. The conductive tracks should be facing upwards when fixing the matrix board to the milling table as shown below in Fig. 5. Only one NC file has been generated for the circular circuit board which performs both the boring and cutting operations using a 3 mm end mill cutting tool.

The power circuit board can be cut using the CNC milling machine, however this is unnecessary, thus the matrix board is scored by running a flat head screwdriver along the surface. The board is then snapped by hand along the score mark. Although the exact dimensions of the power circuit board are not critical, the size of the board should be approximately 50 mm × 50 mm. Despite opting against generating CAD models for the power circuit board, it is important that the holes drilled in the board align with the tapered rivet plugs in the electronic circuit holder. The hole centres are located 40 mm × 40 mm from one another in a square layout. These 3 mm holes are produced using a pillar drill.

A crimped wire assembly is required to connect the BLDC motor pin terminals to the corresponding power and signal sources. In absence of the semi-automatic crimping machine (AP-K2N) recommended to attach the crimp terminals to 26 AWG wires, an alternative crimping technique is implemented using a needle nose pliers.

1. Cut the stranded 26 AWG wires to 170 mm in length and strip 7 mm and 2 mm of insulation material at the ends of each wire as depicted in Fig. 6a.

**Fig. 6:**
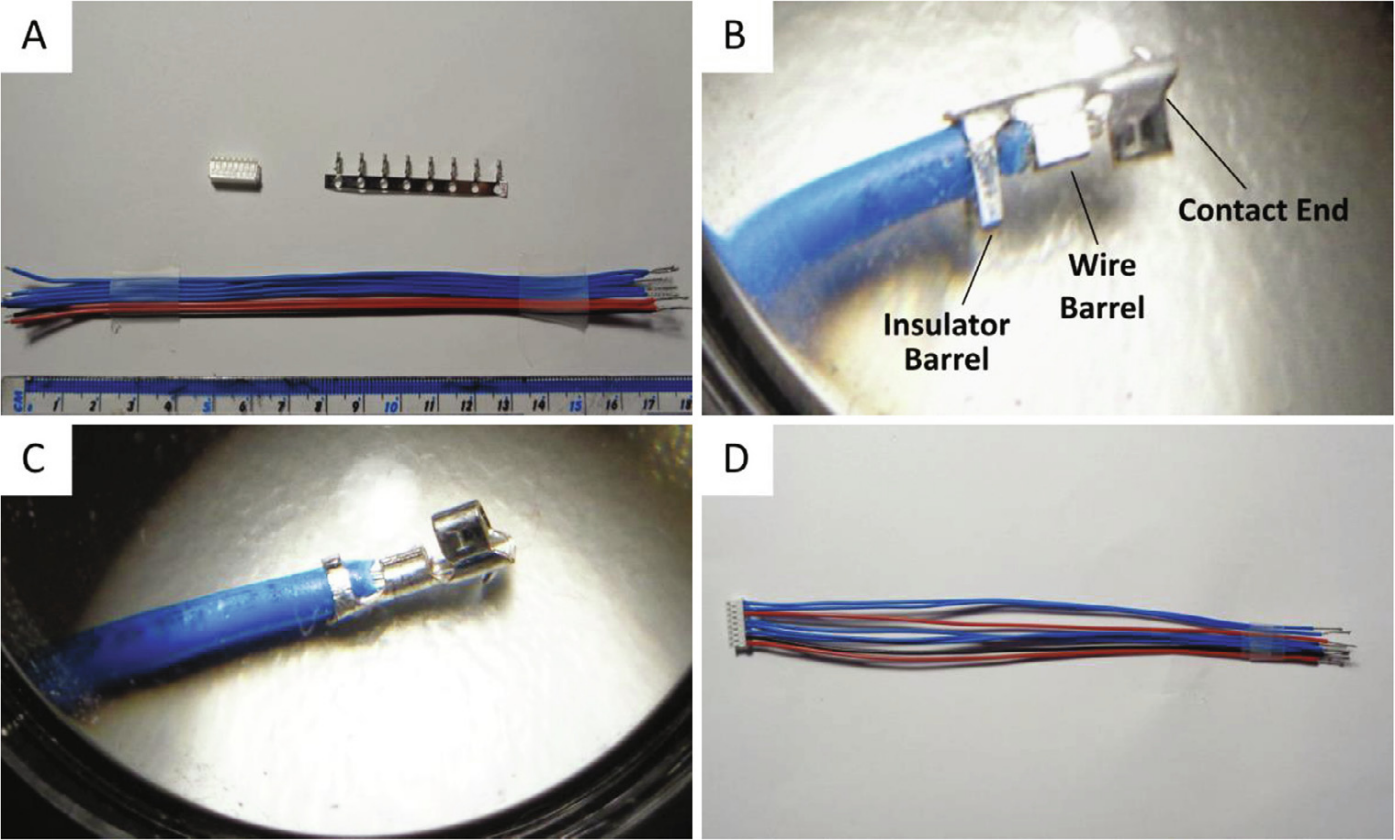
Wire crimping using needle nose pliers. (A) The connector housing, connector crimp terminals and the stranded 26 AWG wires. (B) Inserting a blue wire into the crimp terminal prior to crimping. The features of the crimp terminal are indicated. (C) A compressed crimp terminal attached to a wire. (D) The completed wire assembly. (For interpretation of the references to colour in this figure legend, the reader is referred to the web version of this article.)
2. Coat the exposed 7 mm end of the wires with solder.
3. Insert the 2 mm end of the wire into a crimp terminal. The insulator material should be within the insulator barrel and the exposed wire should be inserted through the wire barrel as shown in Fig. 6b. Using a magnifying glass to view the position of the insulation material and wire in reference to the crimp terminal barrels is recommended.
4. Pinch the insulator barrel around the insulator material using the needle nose pliers. The prongs of the barrel should wrap around the insulator to form a seemingly closed barrel.
5. Next, pinch the tip of the wire barrel prongs together using the needle nose pliers and then flatten the prongs by pressing the tips of the prongs and the underneath of the crimp terminal. Slight adjustments might be required to ensure the shape of the barrel resembles that shown below in Fig. 6c.
6. Ensure the original straight shape of the crimp terminal prior to crimping is retained. If the crimp terminal is slightly bent, the needle nose pliers can be used to delicately straighten it. However, the crimp terminal should be replaced if either the insulator barrel, wire barrel or the contact end becomes damaged.
7. Repeat the above steps for the remaining wires.
8. Insert each of the crimped wires into the connector housing. The blue wires should transmit the control and encoder signals, the red wires should carry the motor and circuit supply voltages and the black wire connects to ground. Insert these wires in accordance with the pinout provided in the BLDC motor datasheet. The wire assembly is shown in Fig. 6d.
9. Tug on each of the wires after inserting them into the connector housing to ensure they are tightly secured.

#### Producing 3D printed parts

**Fig. 7:**
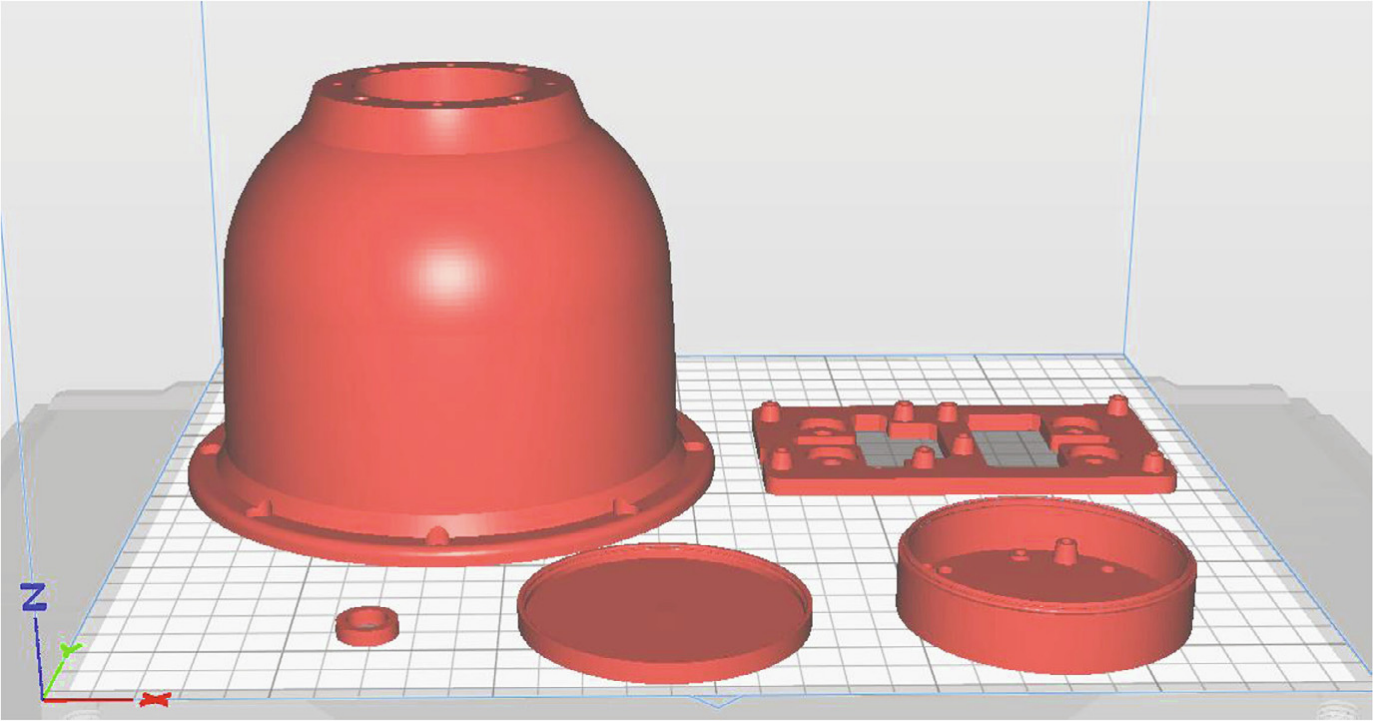
Part layout on the 3D printer bed captured from the Qidi Print software workspace.

The dome structure, battery circuit housing and housing lid, electronic circuit holder and spindle ring can be produced from PLA filament using an FDM 3D printer. The parts are specifically orientated on the printer bed to ensure functional features, such as the snap fit connection between the circuit housing and lid, are not compromised by the unnecessary addition of structural support material. The orientation of the components is shown below in Fig. 7. It is critical that the printer bed is optimally levelled to preserve the dimensional symmetry of the dome housing structure. Bed levelling should be carefully performed prior to printing and the first layers should be inspected during printing to ensure an even distribution of material.

**Fig. 8:**
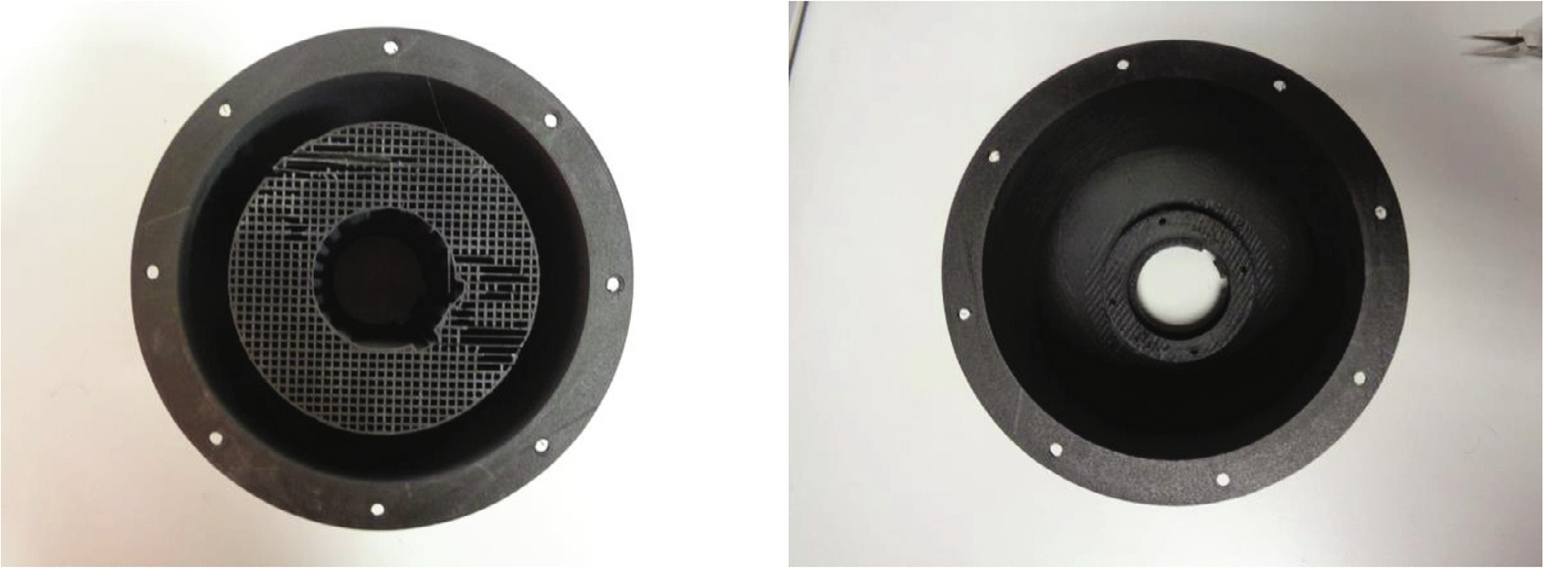
Before and after the removal of the support material from inside the dome housing.

To minimise the mass of the dome structure and thus with the intention of attenuating vibrations during device operation, in addition to reducing the load on the motor, an infill of 15 % is applied. The same infill setting is applied to the smaller parts which require little support during the printing process in contrast to the significant support structure produced during the printing of the dome housing. Following printing, completely removing the internal support can be quite challenging due to limited access to the surfaces to which the support is connected. A pick set and a long nose pliers can be used to remove the remaining material to ensure the LEDs can be installed into the dome shelf during assembly. The dome housing structure is depicted in Fig. 8 with support material attached to its internal surface and subsequently following the removal of the support.

The spindle plate is produced using an SLA printer. The top flat surface of the spindle plate is oriented against the printer build tray and therefore is the first layer printed. The spindle ring was created as a separate part rather than integrated into the spindle plate to reduce the amount of support material required during the build. This reduces the overall cost of the spindle plate and it can be bonded to the spindle plate using super glue. The support structure that is used during the printing process is removed using a high-pressure washing system. Note that the VeroBlack™ material is quite rigid and therefore the spindle plate should be handled with care to ensure the shaft clamp is not damaged.

### Assembly

**Fig. 9:**
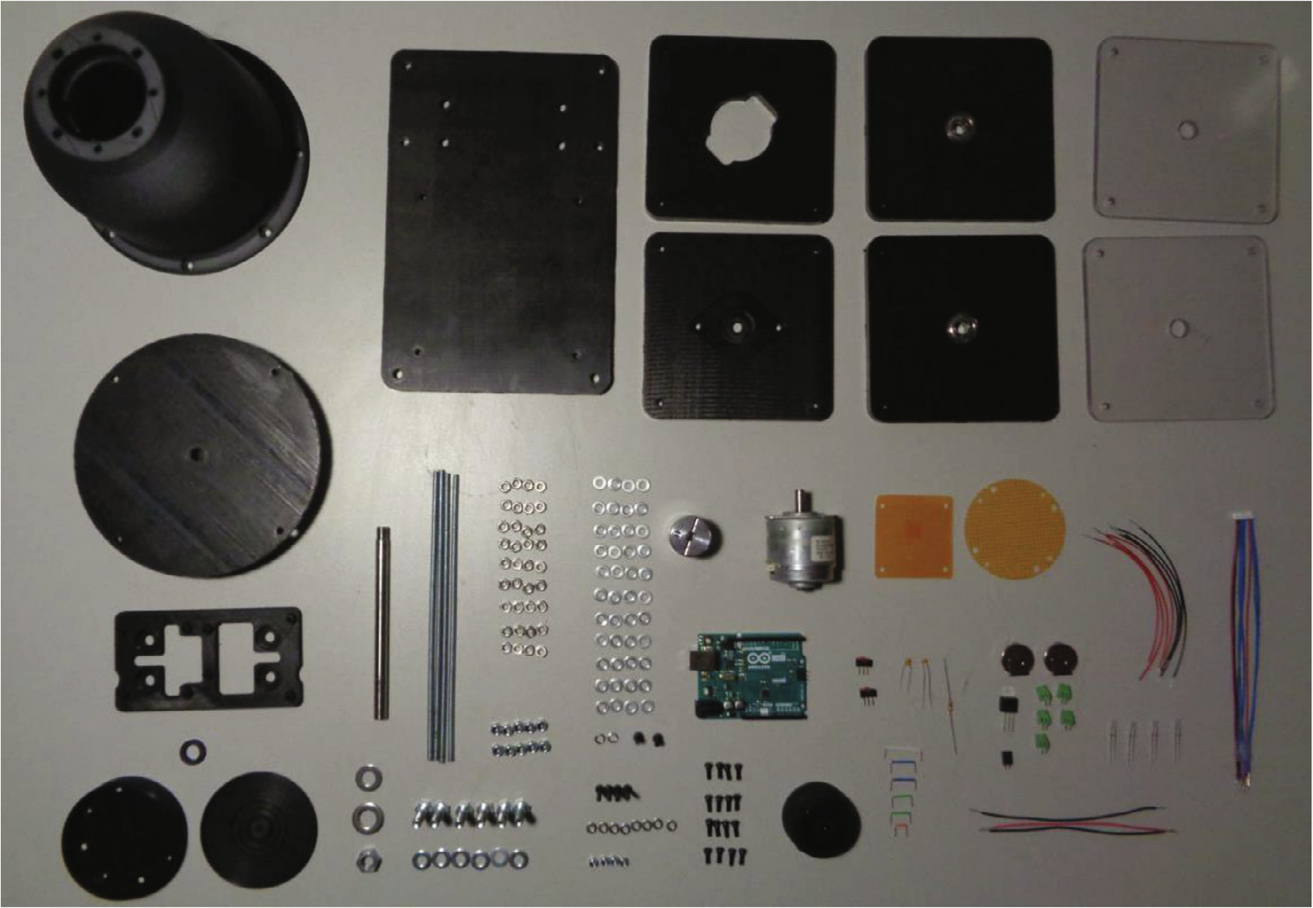
Components and fasteners used in the device assembly.

The assembly involves soldering electronic components to the circuit boards, soldering wires to the LEDs followed by their subsequent installation into the LED shelf within the dome housing, wiring up the Arduino^TM^ Uno and the physical assembly of the device on the optical table. All of the device components and fasteners are displayed below in Fig. 9.

#### Circuitry and LED installation

**Fig. 10:**
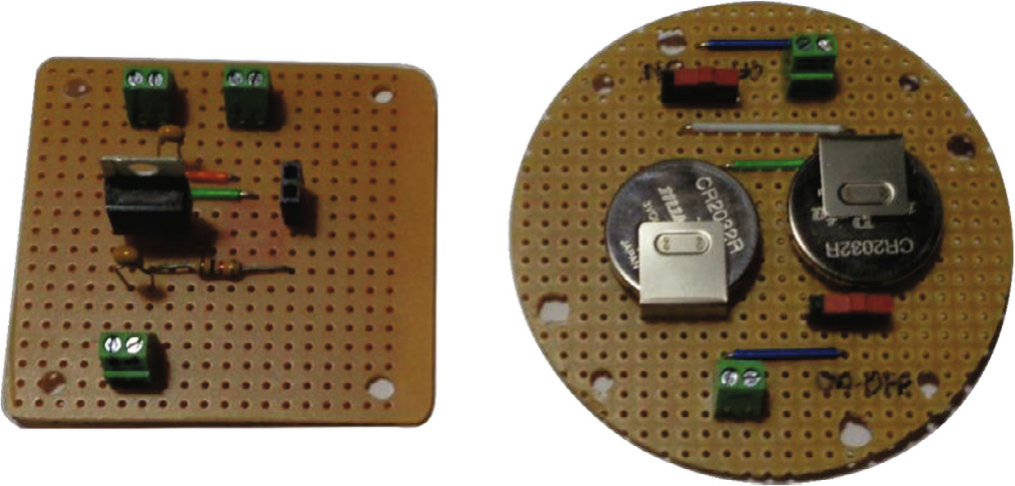
Power and battery circuit boards containing soldered components.

Both the power circuit and the battery circuit schematics are provided in supplementary data 1. Refer to these when soldering the components to the circuit boards. The underside of the battery circuit board may need to be scored at certain locations to break the conductive tracks. Note that the battery circuit component layout is purposely symmetric to minimise any potential imbalances. Both circuits will also require a small amount of additional wire to connect some of the components, as displayed in Fig. 10 below.

Prior to device assembly, the LEDs must be installed in the LED shelf.

1. Strip the insulation material from the ends of the 22 AWG 120 mm red and black wires and apply solder to tin the stranded ends.
2. Solder the wires to the positive and negative terminals of the LEDs. The wires should be soldered near the LED base as depicted in Fig. 11a.

**Fig. 11:**
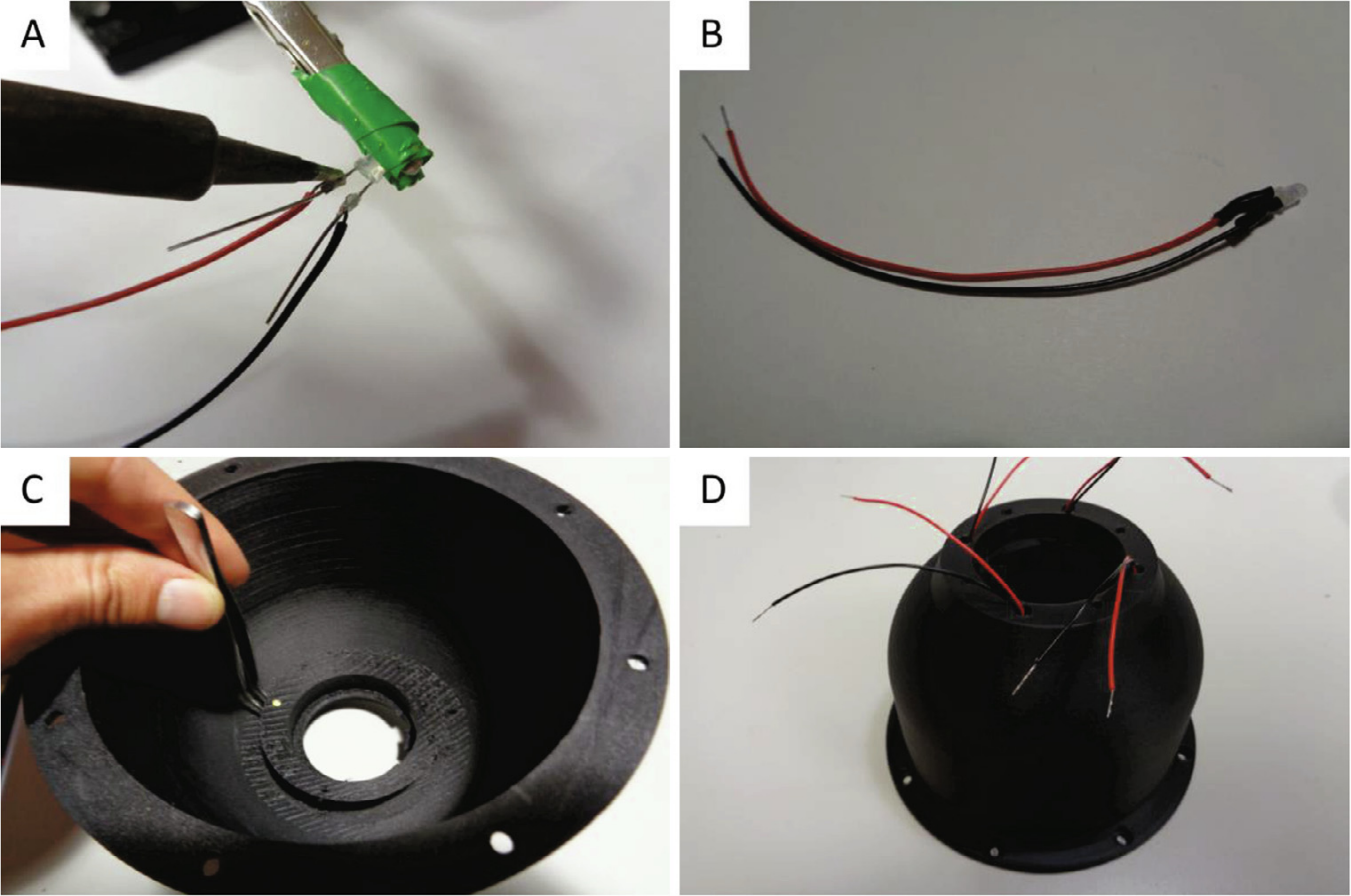
Dome housing LED installation. (A) Soldering the wires to the LED terminals. (B) LED and wires prior to insertion into the dome housing. (C) Using a bent tip tweezers to press the LED through the slot in the shelf. (D) The dome housing with four LEDs installed.
3. Cut and shorten the LED terminals to approximately 8 mm in length.
4. Wrap the LED terminals with 10 mm of heat shrink and apply hot air from a heat gun.
5. Feed the LED wires through the 4.5 mm holes in the flat top surface of the dome housing.
6. Using a bent tip tweezers, carefully insert the LEDs into the slots in the shelf as shown in Fig. 11c. The LEDs should be pressed into the shelf and retained due to the tight fit. If it is too difficult to insert the LEDs, the hole size can be enlarged slightly using a circular needle file and glue can be applied to secure the LEDs within the shelf.

#### Assembling the device

The wireless camera imaging system is assembled from the base layer upwards before being secured to the optical table. A Vernier callipers should be used to verify the position of each layer along the support rods. The layer height positions can be referred to in the “Wireless Camera System Assembly” CAD file. Plain washers (M4) are placed between each acetal layer surface and the fastened hex nuts (M4). Hence, the use of washers is not repeatedly mentioned in the assembly procedure which is outlined below:

1. Attach two nuts (M4) to each of the support rods approximately 50 mm along the rods and slide the rods through the stabiliser layer. Ensure a plain washer is placed on the support rods before sliding on the stabiliser layer.
2. Attach another two nuts at the shorter end of each support rod as shown below in Fig. 12.

**Fig. 12:**
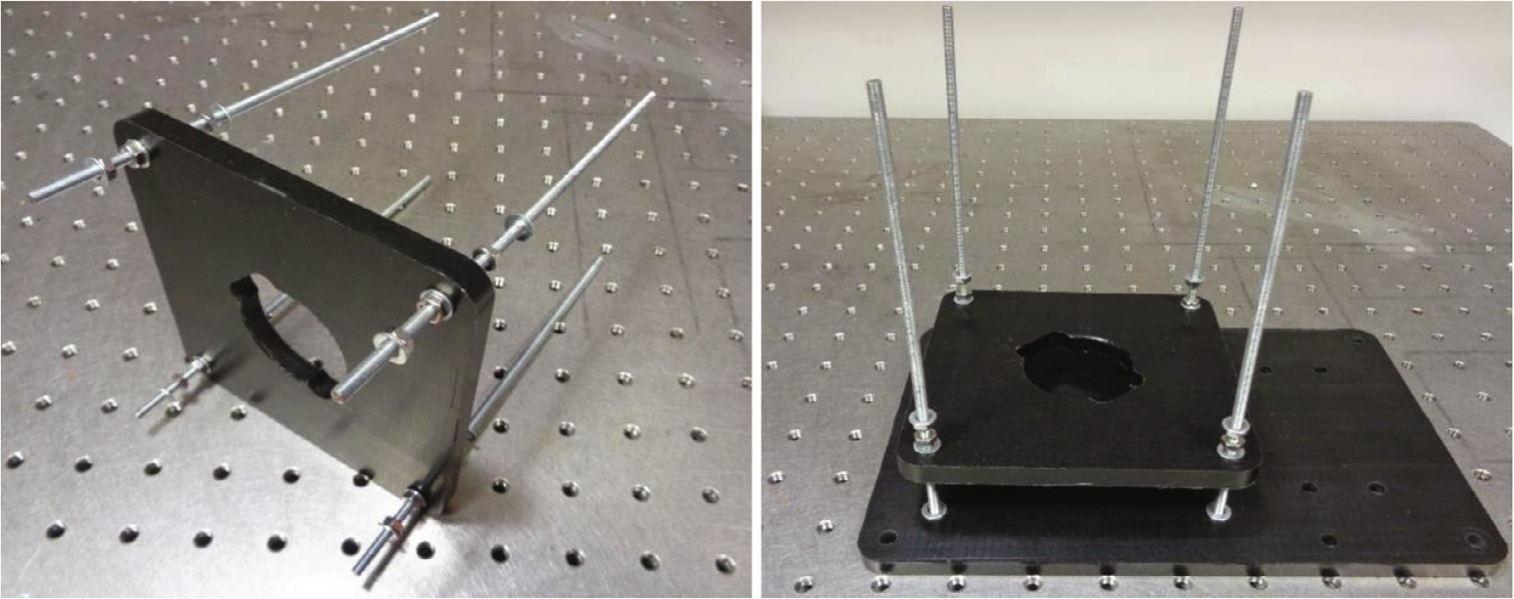
Assembling the lower layers. (Left) Inserting the support rods through the stabiliser layer corner holes. (Right) Securing the support rods to the base layer.
3. Screw the rods through the four holes in the base layer and attach lock nuts to the support rods within the hole cavities on the underside of the base layer.
4. Using a pliers or a spanner, tighten the hex nuts at the base layer surface to secure the support rods. Also tighten the hex nuts either side of the stabiliser layer.
5. Adjust the height of the stabiliser layer so that it is approximately 25 mm from the surface of the base layer as shown in Fig. 12. Ensure the layer is level by using a spirit level or measuring the height at each of the corners from the base layer using the Vernier callipers.
6. Secure the BLDC motor to the motor mount layer using the 16 mm socket screws, ensuring to use spring washers in addition to plain washers.
7. Slide the motor mount layer along the support rods and carefully press the BLDC motor through the opening in the stabiliser layer. The BLDC motor connector should align with the notch in the opening and be directed towards the area of the base layer where the electronics will be positioned.
8. Secure the motor mount layer approximately 17 mm from the stabiliser layer by tightening the nuts either side of it. Attach the shaft coupling to the motor shaft and tighten the grub screw using a hex key as shown in Figs. 13 and 12.

**Fig. 13:**
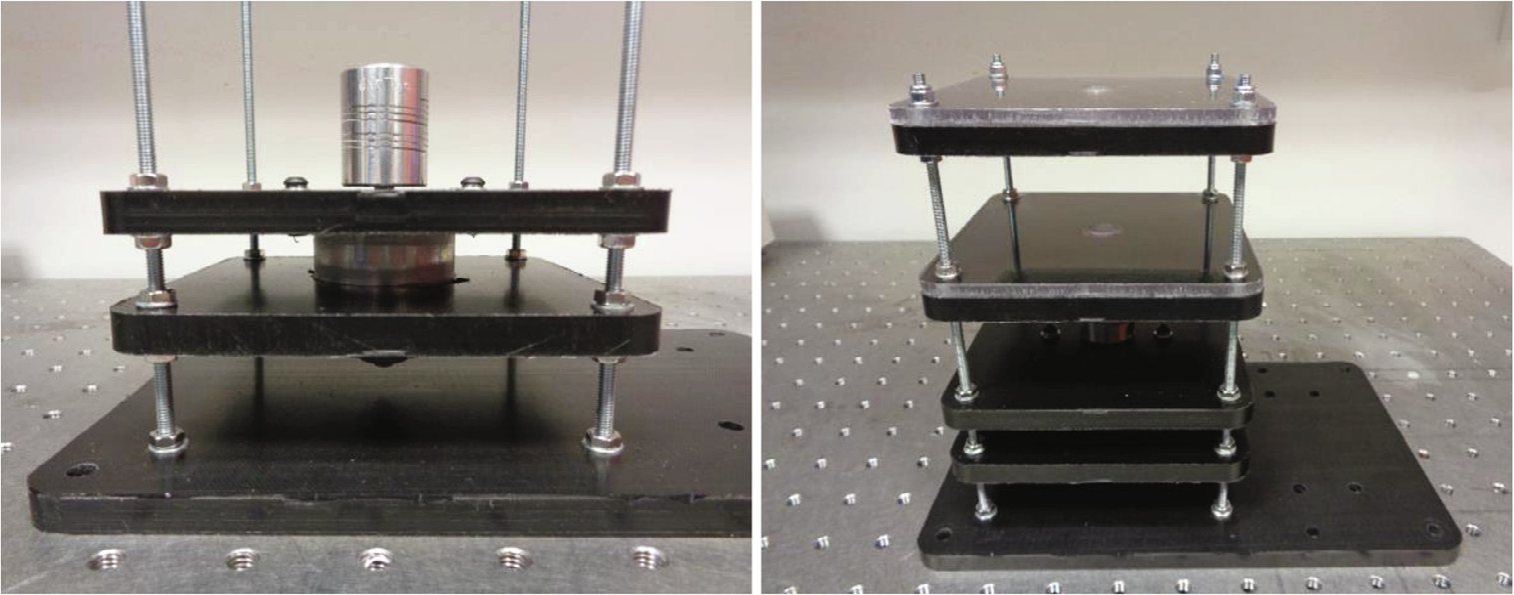
Mounting the BLDC motor and assembling the top layers (Left) The motor mount layer is fastened to the support rods with the BLDC motor secured and inserted through the stabiliser layer. The flexible coupling is fixed to the motor shaft by tightening the grub screw. (Right) All of the layers are secured to the support rods.
9. Attach another hex nut to each of the support rods and slide the reference bearing and acrylic layers down to the nuts. The bottom surface of the reference bearing layer should be positioned approximately 37 mm from the top surface of the motor mount layer. The acrylic layer should enclose the radial ball bearing.
10. Secure the reference bearing and acrylic layers by attaching and tightening hex nuts at the top surface of the acrylic layer.
11. Another hex nut should be attached to each support rod before the anchor bearing layer and the second acrylic layer are mounted within the assembly. The gap between the bottom surface of the anchor bearing layer and the top surface of the acrylic layer below should be 42 mm.
12. Using a socket wrench, tighten lock nuts (M4) at the top surface of the acrylic layer to fasten the anchor bearing and acrylic layers to the support rods as depicted in Fig. 13.
13. Insert the spindle shaft through the spindle plate bore. The entire threaded section of the spindle shaft should protrude beyond the spindle plate ring.
14. Tighten the clamp around the spindle shaft by fastening the four socket screws (M3) using lock nuts (M3). Ensure the plain washers (M3) are placed on the socket screws at either side of the clamp face. Be careful not to overtighten the lock nuts which could result in fracturing of the clamp.
15. Slide the spindle shaft through the radial bearings and into the flexible coupling as shown in Fig. 14. Tighten the second grub screw in the flexible coupling to lock the spindle shaft in place. Note that mechanical grease might be required to assist sliding the spindle shaft through the radial bearings.

**Fig. 14:**
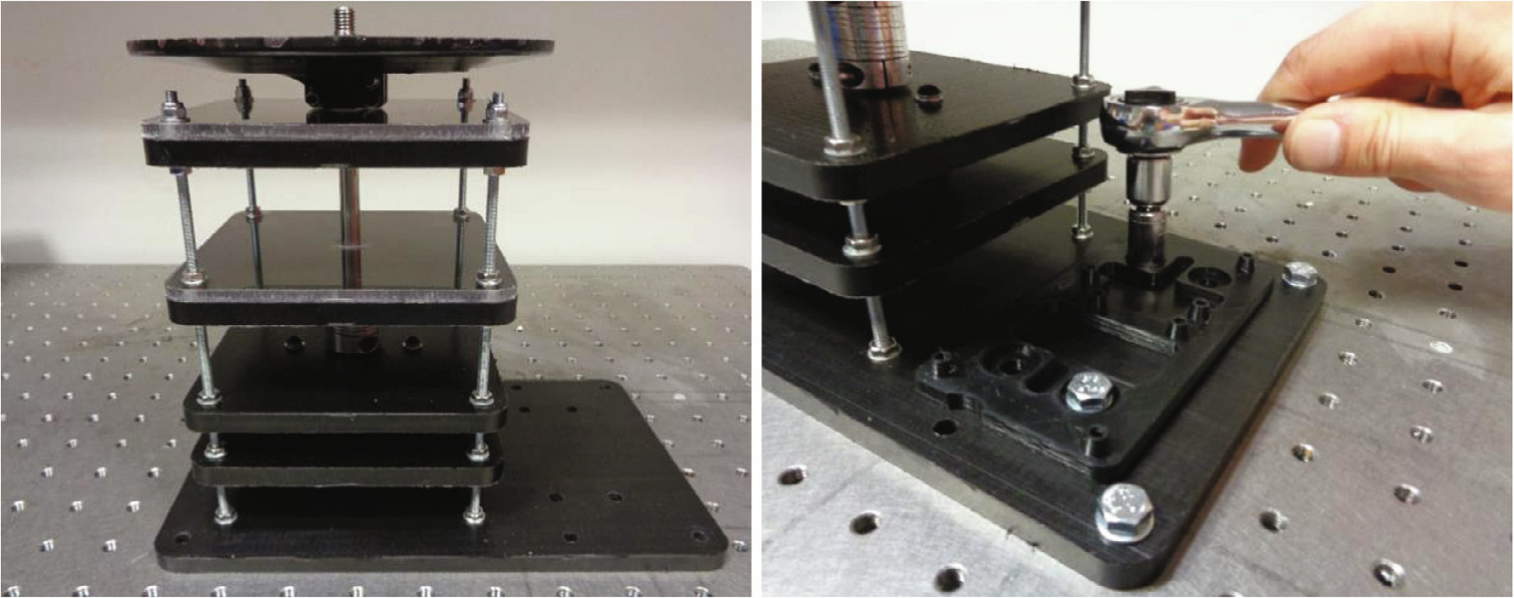
Spindle shaft insertion and fixing the device to the optical table. [Left] The spindle shaft is inserted through the radial bearings and fixed at the shaft coupling. The spindle plate is clamped to the shaft. [Right] Tightening the hex bolts to secure the electronic circuit holder. The base layer is fixed to the optical table.
16. Fasten the device to the optical table using hex bolts (M6).
17. The hex bolts should also be used to secure the electronic circuit holder to the base layer as shown below in Fig. 14.
18. Using a tweezers, insert the cable assembly into the BLDC motor connector housing.
19. Mount the Arduino^TM^ microcontroller and the power circuit board by inserting the snap rivets into the tapered rivet plugs on the circuit holder.
20. Refer to the “Wifi_Camera_System_Control” script (pin assignment) and Fig. 15 below when wiring the BLDC motor to the Arduino^TM^ microcontroller and power circuit board. The wires in the assembly can be labelled to make them easier to identify.

**Fig. 15:**
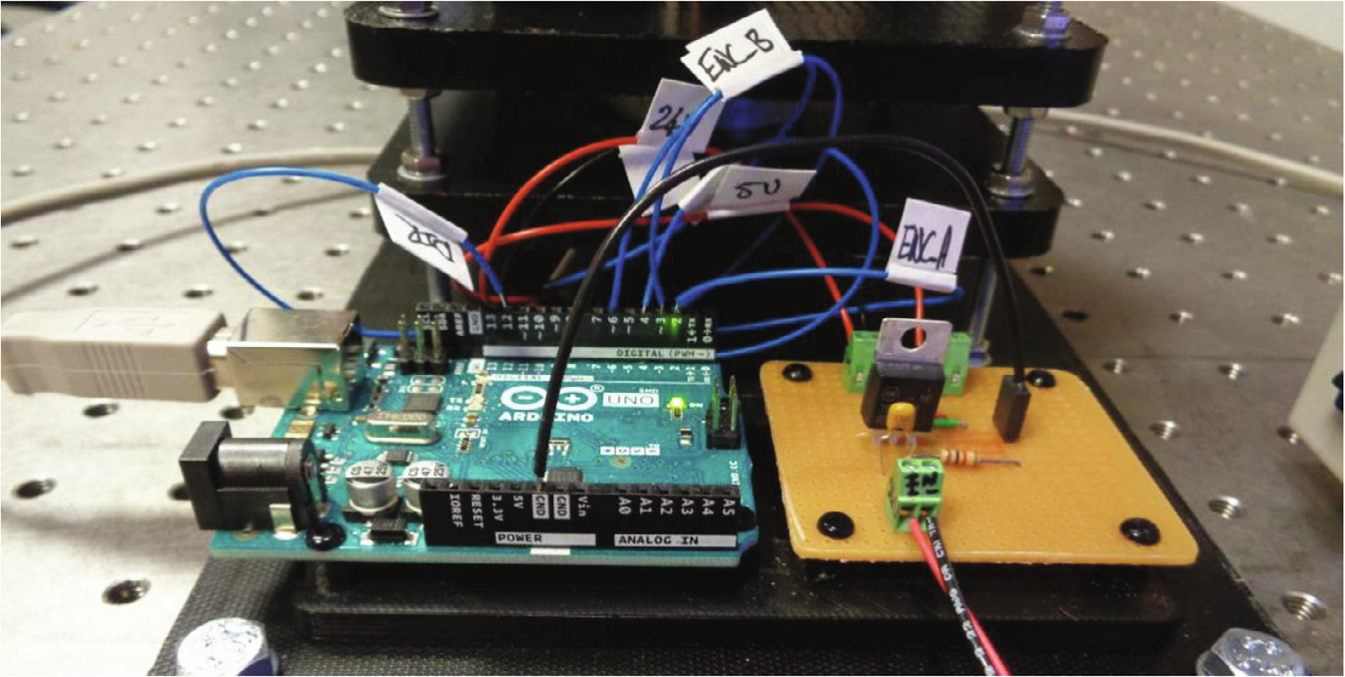
Connections for the BLDC motor to the Arduino^TM^ microcontroller and the power circuit board. Labelled voltage supply wires are connected to the power circuit board. The datasheet for the BLDC motor contains the pinout and the “Wifi Camera System Control” script declares the corresponding connection pins on the Arduino^TM^.

## Operation instructions

**Fig. 16:**
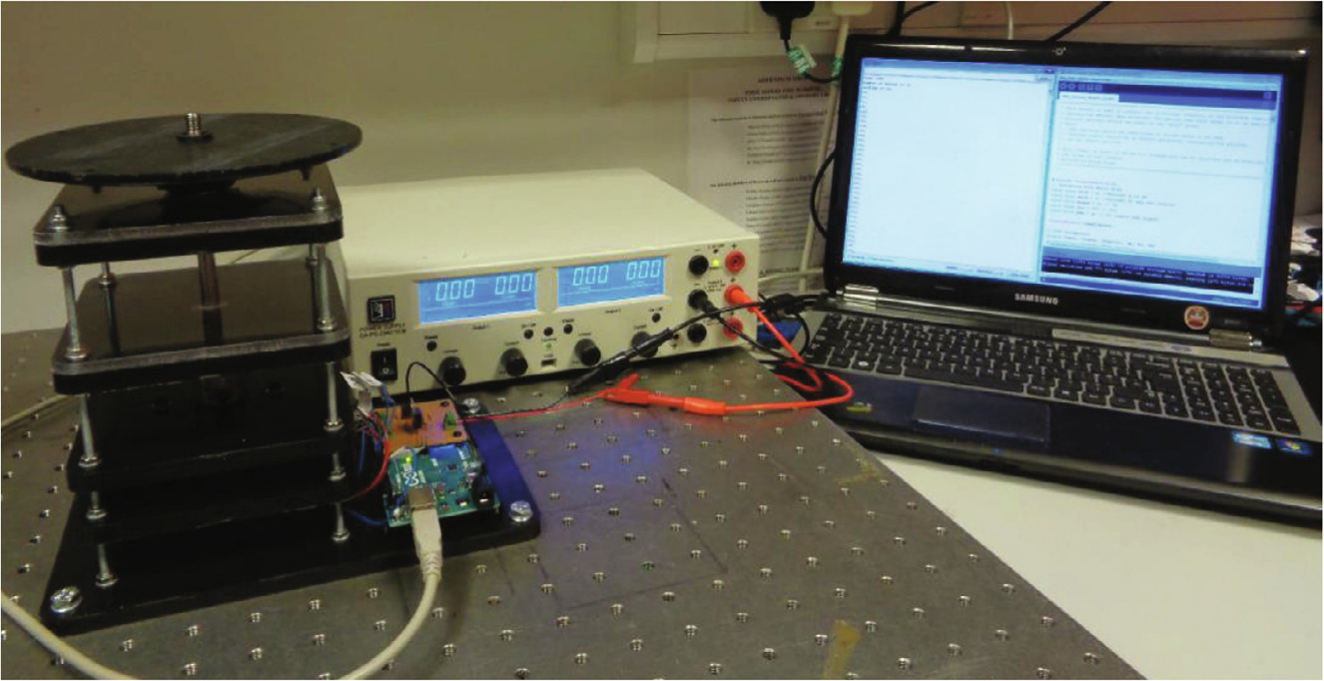
Wireless camera imaging device connected to a power supply and the Arduino^TM^ IDE on a laptop.

The spindle rotation is controlled through the Arduino^TM^ integrated development environment (IDE) on a PC and a power supply is required to provide the input voltage (24 V) for the power circuitry. Fig. 16 depicts the power supply connected to the circuitry and the Arduino^TM^ connected to the laptop. The speed profile can be adjusted from within the “Wifi_Camera_System_Control” script prior to uploading it to the board. This script contains comments to guide the user in adjusting the period and magnitude of the spindle velocity. Moreover, guidelines on how to implement specialised functions such as shake-mode mixing regimes and reversing the direction of spindle rotation are also included within the script. A standard USB Type A/B cable is used to connect the microcontroller to the PC. This also allows the spindle rotational velocity to be viewed and recorded in the serial monitor window.

### Camera assembly

To visualise a LoaD during device operation, the camera assembly must be secured to the spindle plate. The camera assembly consists of the dome housing, the wireless camera, the battery circuit housing and lid and the battery circuit board. The camera battery lifetime is approximately 45 min. The wireless camera must be removed from the seat when it is being charged and consequently, the circuitry and housing must also be detached from the dome structure. Before fastening the dome housing structure to the spindle plate, the LoaD should be secured to the spindle plate using fasteners (M8 & M10 washers and M8 hex nut) and the camera assembly should be constructed. Below are instructions for constructing the camera assembly:

1. Turn on the camera and connect directly to its network.
2. Open the P2PLiveCam app and verify that the camera output can be accessed. The camera should be previously registered on the app before beginning the construction of the camera assembly.
3. Firmly press the camera into the seat of the dome housing.
4. Feed each pair of LED power wires through the 4.5 mm holes in the base of the battery circuit housing.
5. Place the battery circuit housing on the top flat surface of the dome and fasten using snap rivets.
6. Feed the LED wires through the 4.5 mm holes in the battery circuit board and secure the circuit board with snap rivets.
7. Connect the wires to the positive and negative terminals of the circuit, flick the slide switches to transmit power to the LEDs and attach the lid to the circuit housing.

**Fig. 17:**
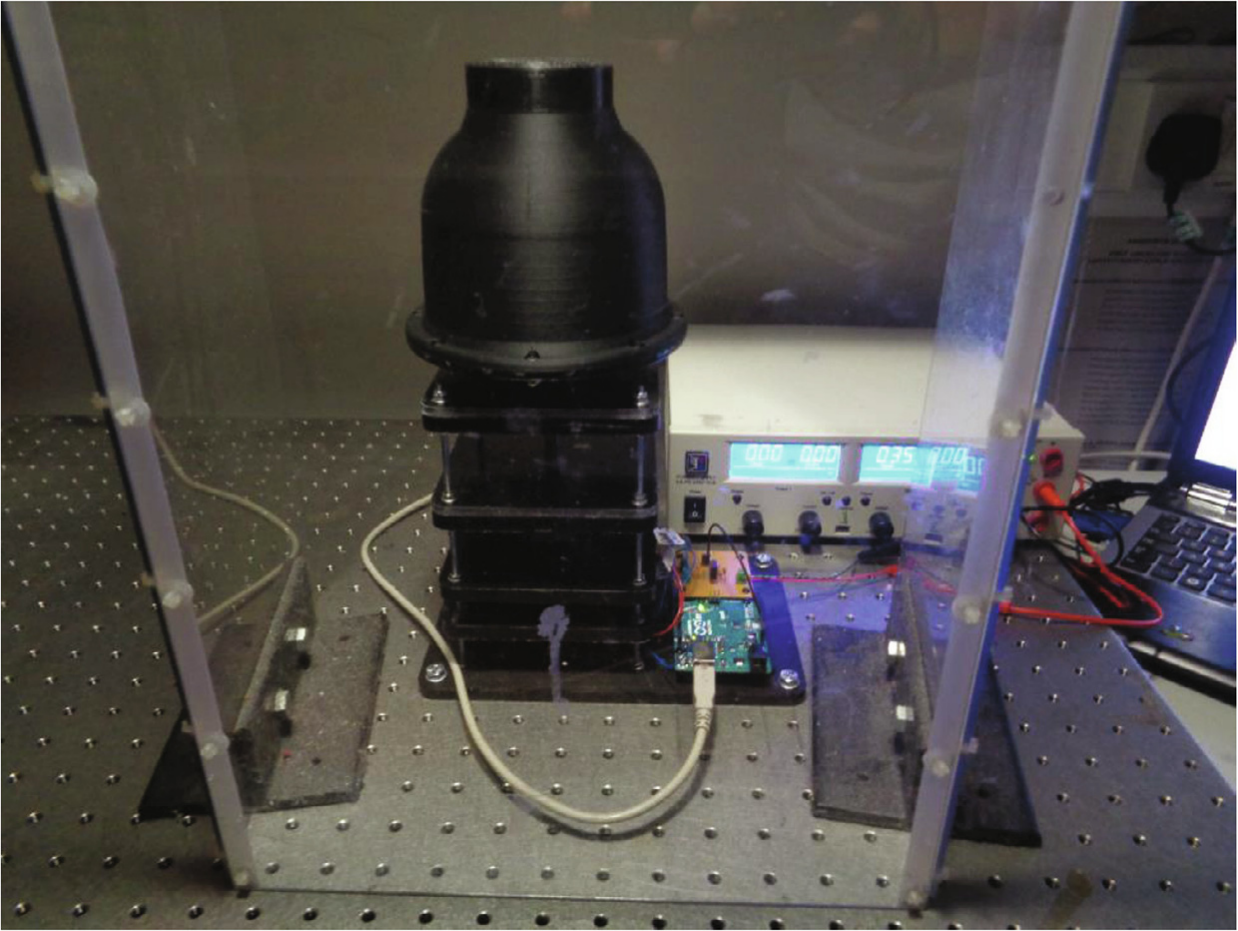
Wireless camera imaging system with the camera assembly mounted and the entire device enclosed by a protective shield.

The camera assembly can be fastened to the spindle plate using fasteners (4x12 mm socket screws and hex nuts (M4)). The device can now be operated by uploading the camera control script and turning on the power supply. The transmitted video stream should be accessed, and the video recording feature activated prior to initiating spindle rotation. Importantly, a protective shield or enclosure should be placed around the system during operation as shown in Fig. 17.

## Validation and characterization

Before recording the operation of a disc and performing colourimetric intensity measurements, the operational speed range of the wireless camera imaging system must be established.

### Vibration analysis

Vibration analysis was conducted to assess the system vibrations at increasing rotational velocities and thus to ascertain the safe operating speed range of the device. In-depth analysis of the vibration frequency components is beyond the scope of this work and the primary intention here is to identify velocity regions of excessive vibrations. Moreover, visual and acoustic observations were made during the vibration trials to correlate acceleration amplitudes with potential destructive vibrations. The spindle plate clamp has been identified as the likely primary point of failure, hence, vibrations should be minimised to prevent component failure and for the protection of the operator. In addition to critical system failure, gradual failure could occur, or the performance of the device could be restricted if the BLDC motor, its internal bearing or the radial ball bearings are damaged by persistently excessive vibrations.

During vibration testing, the rotational velocity of the spindle plate was increased continuously up to 5,000 RPM. This was implemented in increments of 200 RPM to circumvent both the dampening effect of the tuned PI controller and the aggressive acceleration experienced without the implementation of the controller. Hence, a relatively linear and conservative velocity profile was observed which allows vibrations at different speeds to be more easily distinguished and gives time to turn off the power supply if the vibrations become excessively violent and potentially destructive. The testing was conducted with only the spindle plate attached to the spindle shaft and was then repeated with the entire camera assembly fastened to the spindle plate. To assess the influence of dome height on the magnitude of vibrations generated during device operation, two additional dome structures of reduced heights were fabricated. For each test run, a microfluidic disc was secured to the spindle plate using a hex nut (M8).

To record the vibrations, an accelerometer (Adafruit MMA8451 breakout board) was mounted to the side of the top two platform layers of the device as seen previously in Fig. 1. This is the most practical position to mount the sensor and should reflect the forces experienced at the spindle plate clamp; however, in some instances, it may not truly be representative of the vibration contribution to component failure. The sensor outputs an I2C signal which was received by the Arduino^TM^ Uno microcontroller and converted into equivalent vibration accelerations every 100 ms. An additional program script was written (Wifi_Camera_System_Control_with_Vibration) to control the spindle rotation whilst processing and recording the data signal generated by the accelerometer.

**Fig. 18:**
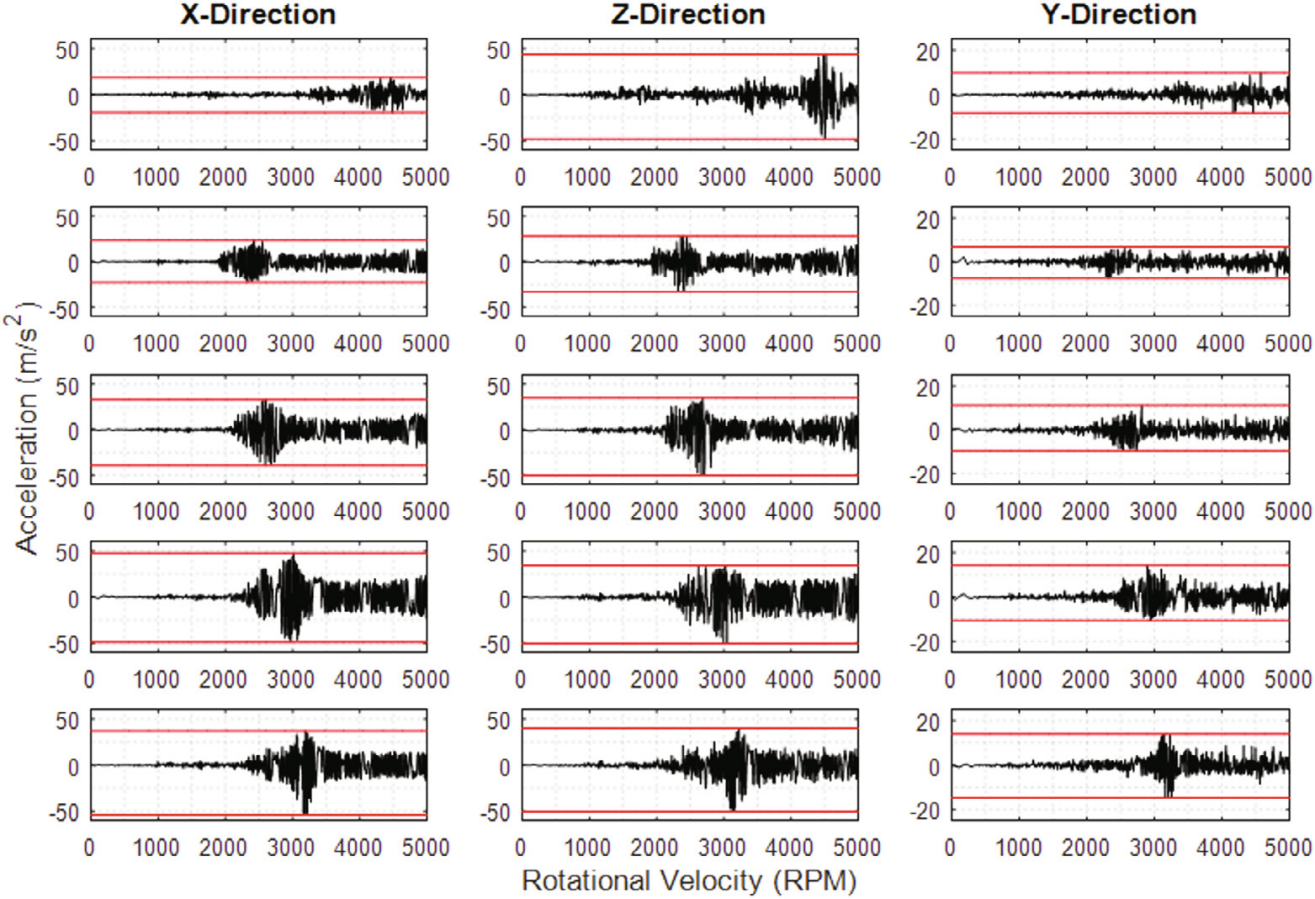
Vibration acceleration generated during device operation for a linearly increasing rotational velocity when the spindle plate and domes of different heights are attached. Data has been recorded using the MMA 8451 accelerometer. Row 1: Spindle plate; Row 2: 85 mm dome camera assembly; Row 3: 65 mm dome camera assembly; Row 4: 50 mm dome camera assembly; Row 5: 85 mm dome structure only. Dome heights are in reference to the distance of the camera lens from the surface of the spindle plate.

Displayed in Fig. 18 below is the raw data of the vibrations acquired in the x-, z- and y-directions during device operation. The reference frame used assigns the y-direction along the axis of rotation and the x- and z-directions radially outwards from the spindle shaft. Specifically, the z-direction pierces through the accelerometer PCB shown in Fig. 1 and the x-direction runs left to right with all directions assumed to intersect at the centre of the spindle shaft. In the x- and z-directions, the maximum acceleration peak magnitudes range from 18 m/s^2^ for the 85 mm camera assembly to over 54 m/s^2^ for the 85 mm dome housing. Evidently, the behaviour of the vibrations in the x- and z-directions is closely related as the majority of vibrations are experienced in these two planes, which encapsulates large components of the rotational vibration forces. Acceleration amplitudes with a magnitude greater than 20 m/s^2^ in the x- and z-directions generate visibly and acoustically violent vibrations. The acceleration amplitudes observed in the y-direction are small in comparison—which is partly due to the limited degree of movement of the device frame in this direction—but in general, exhibit similar characteristics to the those recorded in the other two directions (.

**Fig. 19:**
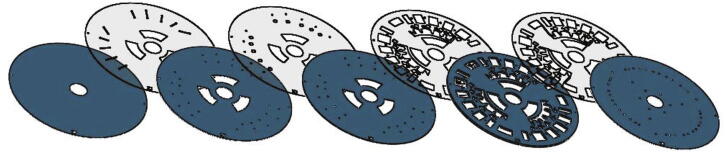
Exploded view of the centrifugal microfluidic disc. The 5 PMMA layers (blue) are arranged from left to right beneath the four PSA layers (translucent). The disc is assembled from the base layer upwards, with the base layer shown on the far left and the vents layer at the far right. (For interpretation of the references to colour in this figure legend, the reader is referred to the web version of this article.)

The vibrations of the device when the unloaded spindle plate is mounted to the shaft generally increase proportionally with the rotational velocity, excluding the reduction after 4,500 RPM in the x-direction and the significant peaks observed in the z-direction. These peaks indicate transient vibration events which may suggest the occurrence of resonant frequencies but do not appear to translate directly to the responses observed when the spindle plate is loaded with the dome structures. Nonetheless, defined periods of excessive vibrations also exist for the loaded responses which may reveal the critical speed of the system for different loads.

A distinct trend exists between the loaded responses which is particularly prevalent in the x- and z-directions. The maximum positive and negative peak amplitudes occur at increasing rotational velocities for lighter loads. This would permit higher speeds to be achieved for these domes without experiencing potentially destructive vibrations. It is quite clear that the presence of the camera assembly produces a dampening effect and thus attenuates system vibrations as there is a significant difference in the acceleration peak magnitudes in all directions between the 85 mm dome camera assembly and the 85 mm dome housing structure. Moreover, beyond 3,500 RPM, the device acceleration amplitudes for all 3 camera assemblies in each direction remains relatively constant with a gradual increase in the vibration intensity towards 5,000 RPM.

Considering the vibration plots for the 85 mm camera assembly, the operating speed range should primarily be confined below 2,200 RPM. However, rotational velocities above 2,750 RPM generate relatively small vibrations that should not significantly compromise the structural integrity of the spindle plate clamp or any other potential points of failure. Hence, rapidly accelerating/decelerating (>30 rad/s^2^) through this resonance zone should minimise the stresses on the system and permit safe operation of the device. Intentionally avoiding this region would have to be considered during LoaD design when employing the wireless camera imaging system for disc inspection. Interestingly, the amplitudes at the critical speeds for the different height camera assemblies appears to decrease with increasing height. The camera assembly with the lowest height profile (50 mm) for instance, generated considerably violent vibrations at the critical speed and sustained excessive system vibrations beyond this region. This may suggest that domes of greater height could be used in the device and as such, support the use of cameras with smaller FOVs. An investigation would have to be conducted however, to establish the performance of the system for elevated dome structures.

### Visualising a microfluidic disc

A microfluidic disc was fabricated to demonstrate the visual inspection capabilities of the wireless camera visualisation system. The stability of on-disc reagent measurements was also investigated.

#### Disc fabrication

A laminated centrifugal disc was assembled using 1.5 mm (RS Ireland), 0.5 mm and 0.375 mm (PLEXIGLAS®) Poly(methyl methacrylate) (PMMA) layers. An Epilog zing CO_2_ 30 W laser engraver was used to cut out the disc features from the PMMA sheets. Pressure sensitive adhesive (PSA) (ARcare 7840, Adhesive Research) was cut using a vinyl cutting plotter (CE6000-40, Graphtec Corporation). An exploded view of the individual PMMA and PSA layers with the cut-out disc features are shown in Fig. 19. Dissolvable film (DF) was used in the fabrication of DF-based speed-regulated burst which was also cut on the cutting plotter. The PMMA layers were assembled on a guide jig and bonded together with the PSA. The DF burst valves were carefully positioned within the disc during assembly. A pneumatic roller-press compressed the PMMA layers together to fortify the bond and effectively ensure the disc was properly sealed.

#### Disc function

The microfluidic disc was designed to handle parallel dilutions of concentrated dyed water. The dyed water represents a reaction product that could be measured using a colourimetric sensing scheme and the dilutions served to demonstrate the ability of the sensing strategy to discern between different colour intensities. The concentrated dye was injected into the loading chamber and the mixing chambers were pre-loaded with DI water saturated with NaCl. Metering chambers facilitated the delivery of variable volumes of concentrated dyed water for subsequent mixing with the NaCl solution. The colour intensity of the diluted dye within the detection chambers is recorded during mixing and a calibration curve is plotted for the final intensity responses. This curve is then compared to the response acquired using a bench top plate reader with the absorbance wavelength set to 415 nm to determine the sensitivity and accuracy of the wireless camera imaging system.

#### Disc visualisation and colour intensity detection

**Fig. 20:**
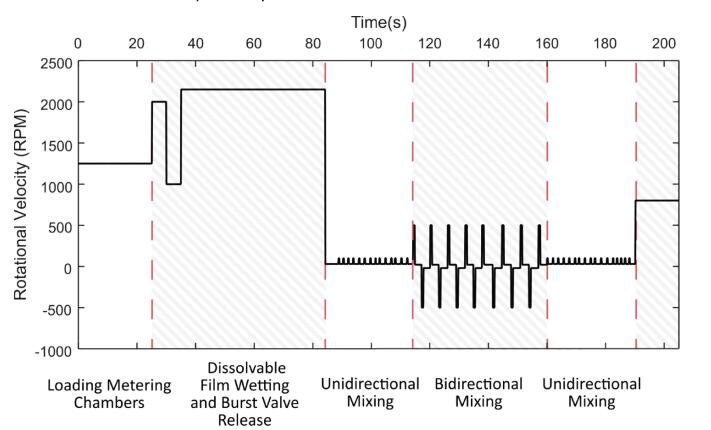
Spin profile executed for the operation of the LoaD. Unit operations are outlined by solid and hatched regions with descriptions provided below the graph.

The disc and the camera assembly were secured to the spindle plate and the spin profile shown below in Fig. 20 was implemented. The operation of the disc requires the burst valve speed to be exceeded to release the liquid from the metering chambers. Furthermore, mixing is achieved by executing the unidirectional mixing and bidirectional mixing functions to generate shake-mode mixing of the liquid in the detection chambers. Both of these stages are apparent on the spin profile, with the unidirectional mixing performed before and after the bidirectional mixing. Due to the load of the camera assembly, the acceleration of the spindle is reduced which in turn, limits the intensity of the forced convection within the detection chambers during mixing. The rotational velocity is increased initially after 25 s to burst the valves and the mixing regime begins 85 s into the spin profile. A 60 s delay exists between ramping the speed up and the shake-mode mixing due to the dissolution time period required to breach the DF barrier.

**Fig. 21:**
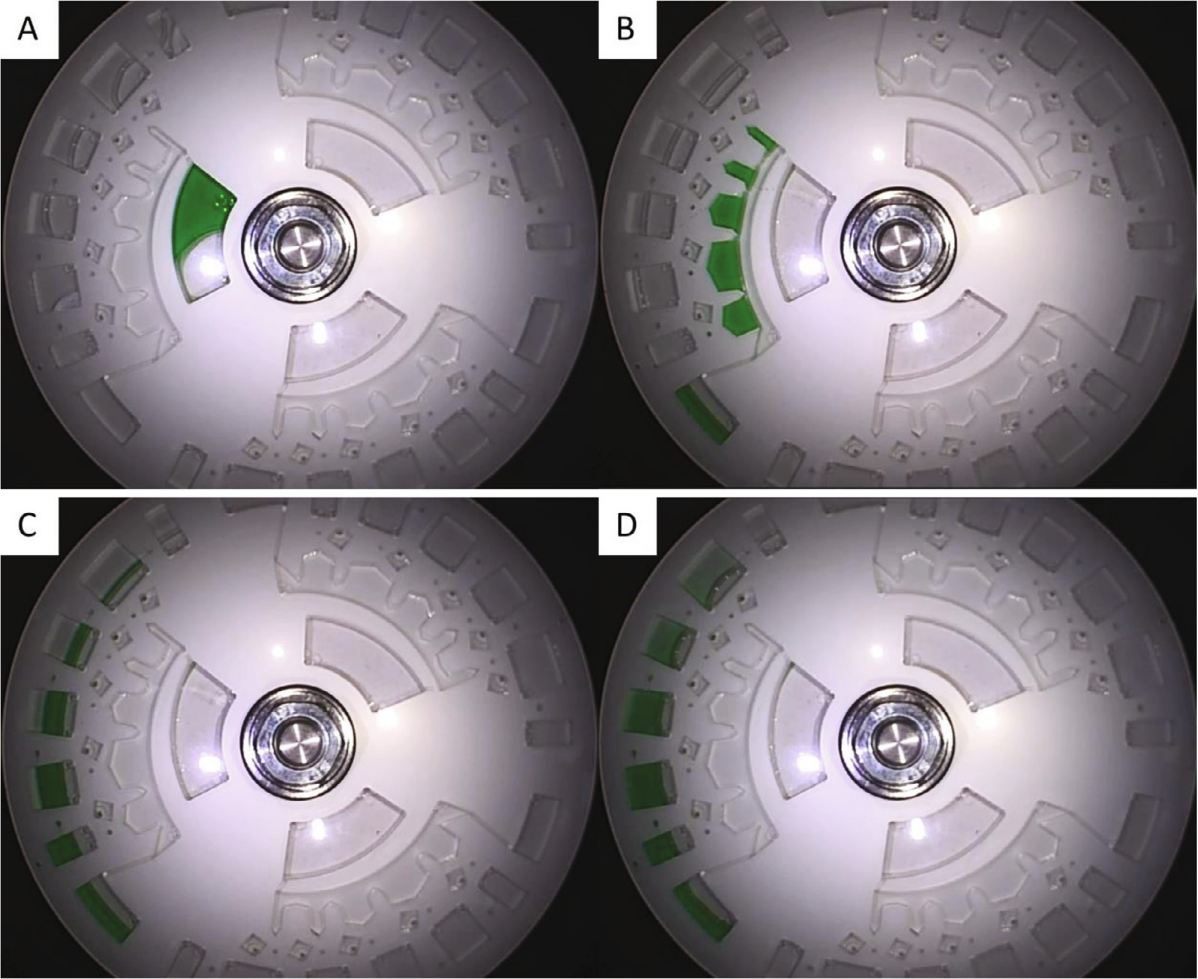
Operation of LoaD for dyed water dilution and transportation to detection chambers. (A) Concentrated dye loaded onto the disc with NaCl saturated DI water pre-loaded in detection chambers (t = 0 s). (B) Loaded dye is metered and excess liquid is directed to the waste reservoir (t = 20 s). (C) Burst valves are breached and liquid is transported into the detection chambers (t = 60 s). The dense NaCl solution remains separate to the dyed water. (D) NaCl solution and dyed water are mixed and distinct colour tones are visible between the detection chambers (t = 210 s).

Still images of the disc at different stages during the spin profile have been extracted from the MP4 video file and are shown below in Fig. 21. The high image quality allows the disc operation to be observed and the individual features to be inspected, even when passing through regions that produce high vibrations and up to spin rates of 5,000 RPM (see supplementary data 2). This is highly beneficial when differentiating between reliable and dysfunctional fluidic structures during disc development. The detection chambers have been placed near the perimeter of the disc to exploit the radially outward pumping generated by the centrifugal forces. Note that the light intensity varies radially due to the illumination by the LEDs. Hence, measurements in multiple detection chambers should occur at an equal distance from the disc centre to maximise uniformity.

**Fig. 22:**
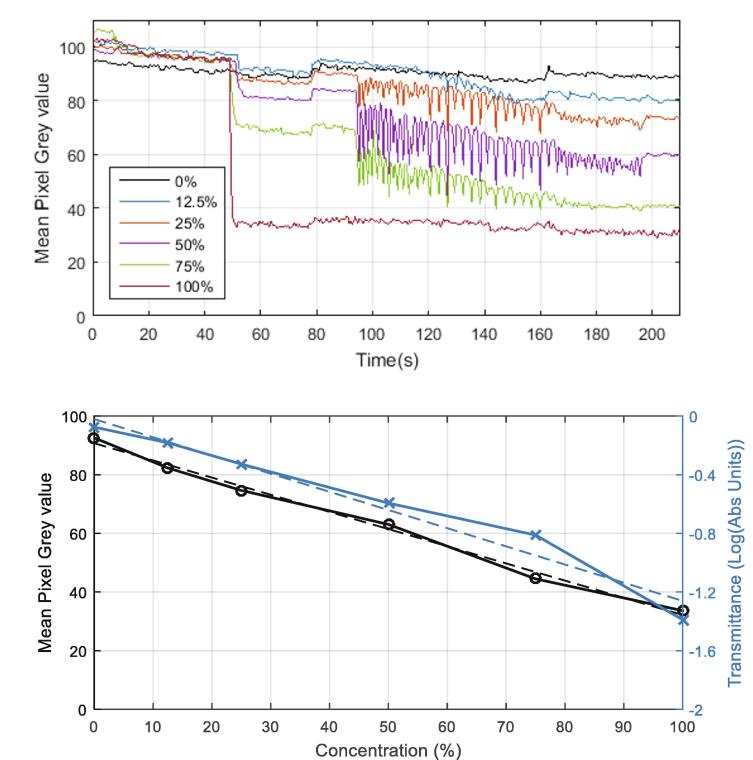
(Top) Real-time colour intensity measurements for varying food colouring concentrations during disc operation. (Bottom) Measurement of the detection chamber liquids at 415 nm on the plate reader (x-marker and right y-axis) and the calibration curve created from the final colour intensity data points (o-marker and left y-axis). Dashed lines are approximate linear trendlines for the overlaid data plots. The experiment was performed in triplicate and the above plots are averages of the three datasets. The mean pixel grey value refers to the averaged greyscale colour intensity (0 – 255) within the region of analysis (see red circles in supplementary data 3) Abs – Absorbance. (For interpretation of the references to colour in this figure legend, the reader is referred to the web version of this article.)

The colour intensity of the liquid in the detection chamber is analysed throughout the operation of the disc as shown below in Fig. 22. In respect to the trajectories of the ‘real-time’ data curves, a notable descent occurs as the spin profile enters the mixing regime. A gradual decline is then observed as the liquids mix. Evidently, this extended mixing period could be somewhat attributed to the limited torque output of the motor in respect to the considerable load of the camera assembly. As a result, the mixing of some of the liquids (25 % and 50 %) may be incomplete and therefore, the final colourimetric data is likely to be slightly inaccurate.

A slight shift in the trajectory of the real-time data plots at 80 s appears to coincide with a brightness adjustment on the video recording (see supplementary data 3). It is unclear whether this is an automated feature of the camera which possibly compensates for inadequate ambient light levels. Without calibrating for the background brightness levels, the automated brightness adjustment could reduce the accuracy and precision of colour intensity measurements when using this camera and thus effectively limit sensitivity. Analysis of the final mixed solutions within the detection chambers has revealed the linear relationship between the dye concentration and the mean pixel grey value, as displayed in the second graph of Fig. 22. These mixed solutions (50 µL) were then extracted from the detection chambers after spinning had ceased, added into a half-area 96-well plate and absorbance measurements were conducted at 415 nm using a plate reader (Biotek ELx808). The transmittance was calculated from the absorbance data to produce a linear response that could easily be compared with the grey pixel data. Evidently, the absorbance response shown below in Fig. 22 exhibits a similar profile to the colour intensity measurements.

## Conclusion

A wireless camera-based flow visualisation system for inspecting the operation of the LoaD devices has been developed and tested. This easily reproducible device was primarily fabricated using a 3D printer and a low-cost CNC milling machine from widely available and inexpensive materials. The safe operation velocity range of the device was established to be within 0–2,200 RPM and 2,750–5,000 RPM. This was determined by evaluating the system vibrations at increasing rotational velocities and identifying potentially destructive vibrations such as those which occurred between 2,200–2,750 RPM. The excessive vibrations observed within this narrow range could possibly be attenuated by implementing slight design alterations such as; increasing the height of the dome structure, ensuring the mating surfaces of the dome and spindle plate are completely parallel or/and by tapering both the spindle shaft and the spindle plate bore to ensure the plane of the plate is orthogonal with the shaft axis.

The wireless camera imaging device was employed to record the operation of a LoaD designed to facilitate the parallel dilution of a dyed water. The system could support real-time colourimetric intensity measurements to discern different dilution concentrations. Additionally, the applicability of this device could potentially be extended to facilitate real-time luminescent detection methods. The physical synchronisation between the spindle plate and the wireless camera ensured a relatively stable image could be recorded and thus, generally permitted the accurate measurement of the liquids. The apparent automated brightness adjustment could limit the overall sensitivity that can be achieved using this system; however, performing a background brightness subtraction to correct for this adjustment should counteract the imprecision introduced by this feature.

## Declaration of Competing Interest

The authors declare no conflicting interests that could have influenced the reporting of work detailed in this paper.
