# Supplementary material for: Design and fabrication of a low-cost wireless camera imaging system for centrifugal microfluidics
Source: HardwareX. 2022 Jan 8;11:e00259. doi: 10.1016/j.ohx.2022.e00259 (PMC9058586; doi:10.1016/j.ohx.2022.e00259)
Supplement: Supplementary data 1 [file mmc1.docx]

**Supplemental Circuit Schematics**


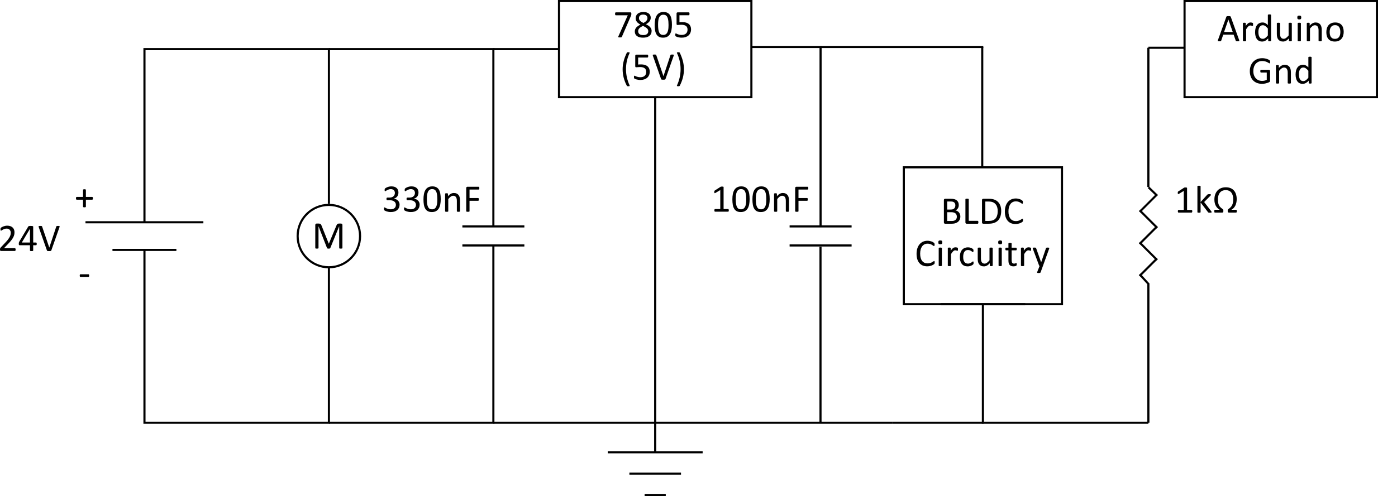


Figure S1. Power circuit schematic


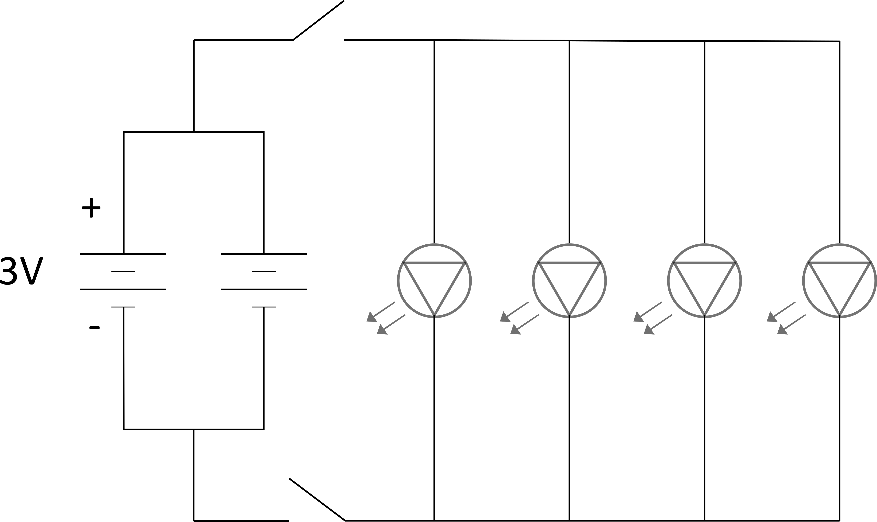


Figure S2. Battery circuit schematic
